# Effects of Maternal Vitamin D Supplementation During Pregnancy and Lactation on Infant Acute Respiratory Infections: Follow-up of a Randomized Trial in Bangladesh

**DOI:** 10.1093/jpids/piab032

**Published:** 2021-07-02

**Authors:** Shaun K Morris, Lisa G Pell, Mohammed Ziaur Rahman, Abdullah Al Mahmud, Joy Shi, Tahmeed Ahmed, Michelle C Dimitris, Jonathan B Gubbay, M Munirul Islam, Tahmid Kashem, Farhana K Keya, Minhazul Mohsin, Eleanor Pullenayegum, Michelle Science, Shaila S Shanta, Mariya K Sumiya, Stanley Zlotkin, Daniel E Roth

**Affiliations:** 1 Centre for Global Child Health, The Hospital for Sick Children, Toronto, Ontario, Canada; 2 Department of Pediatrics, University of Toronto and The Hospital for Sick Children, Toronto, Ontario, Canada; 3 Child Health Evaluative Sciences, The Hospital for Sick Children, Toronto, Ontario, Canada; 4 Division of Infectious Diseases, The Hospital for Sick Children, Toronto, Ontario, Canada; 5 Infectious Diseases Division, International Centre for Diarrhoeal Disease Research, Bangladesh, Dhaka, Bangladesh; 6 Nutrition and Clinical Services Division, International Centre for Diarrhoeal Disease Research, Bangladesh, Dhaka, Bangladesh; 7 Department of Epidemiology, Harvard University T H Chan School of Public Health, Boston, Massachusetts, USA; 8 Public Health Ontario, Toronto, Ontario, Canada; 9 Primary and Community Health Branch, Ministry of Health, Edmonton, Alberta, Canada; 10 Division of Biostatistics, Dalla Lana School of Public Health, University of Toronto, Toronto, Ontario, Canada

**Keywords:** acute respiratory tract infection, Bangladesh, infant, influenza, pregnancy, respiratory syncytial virus, vitamin D

## Abstract

**Background:**

We examined the effect of maternal vitamin D supplementation during pregnancy and lactation on risk of acute respiratory infection (ARI) in infants up to 6 months of age in Bangladesh.

**Methods:**

This study was nested in a randomized, double-blind, placebo-controlled, 5-arm dose-ranging trial of prenatal and postpartum vitamin D supplementation. One group of women received 0 IU vitamin D per week during pregnancy and for 26 weeks post delivery (“placebo” group), one group received high-dose prenatal vitamin D supplementation of 28 000 IU per week and 26 weeks post delivery, and there were 3 additional dose-ranging groups receiving vitamin D supplementation during pregnancy only (4200, 16 800, and 28 000 IU per week, respectively). Episodes of ARI were identified by active and passive surveillance. The primary outcome was microbiologically confirmed ARI, and the primary analysis compared the high-dose prenatal plus postpartum vitamin D vs placebo groups.

**Results:**

In total, 1174 mother-infant pairs were included. Among infants born to mothers in the placebo group, 98% had a venous umbilical cord 25(OH)D level below 30 nmol/L compared with none in the high-dose prenatal plus postdelivery vitamin D group. Incidence of microbiologically confirmed ARI in the high-dose prenatal plus postpartum vitamin D (1.21 episodes per 6 person-months; N = 235) and placebo groups (1.07 episodes per 6 person-months; N = 234) was not significantly different (hazard ratio of 1.12 [95% confidence intervals: 0.90-1.40]). There were no differences in the incidence of microbiologically confirmed or clinical ARI, upper, lower, or hospitalized lower respiratory tract infection between high-dose prenatal plus postpartum vitamin D and placebo groups.

**Conclusions:**

Despite a high prevalence of maternal baseline vitamin D deficiency and significant effects of maternal vitamin D supplementation on infant vitamin D status, the intervention did not reduce the risk of microbiologically confirmed ARI in infants up to 6 months of age.

Acute respiratory infections (ARIs) are a major cause of childhood morbidity and mortality globally [1]. In children, upper respiratory tract infections (URTI) frequently precede more severe lower respiratory tract infections (LRTI). In 2017, over 800 000 deaths among children under the age of 5 years were estimated to be attributable to LRTIs, with LRTI-associated deaths being disproportionately higher in South Asia and sub-Saharan Africa [1, 2].

Respiratory syncytial virus (RSV) and influenza are among the most common and important viral causes of childhood respiratory infection. In 2015, RSV caused an estimated 30 million (95% confidence interval [CI]: 19-47) cases of LRTI and 118 200 (95% CI: 94 600-149 400) deaths in children under 5 years in low- and middle-income countries (LMICs) [3]. Influenza has been estimated to cause more than 45 000 annual deaths among children younger than 5 years, almost all of which occurred in LMICs [4]. Since treatment of RSV, influenza, and other viral respiratory infections remains suboptimal, prevention strategies are critical toward improving child survival.

Evidence suggests that vitamin D may play a role in modulating the immune response to ARI. Vitamin D has been shown to induce reactive oxygen and nitrogen intermediaries [5, 6], stimulate the proliferation of monocytes [7], promote phagocytosis [8], and activate transcription of antimicrobial peptides, such as cathelicidin and defensins [9–11], potentially conferring antiviral effects. Vitamin D also inhibits T and B cells and shifts the immune response toward an anti-inflammatory state [12].

Observational studies showed that lower concentrations of serum 25-hydroxyvitamin D (primary biomarker of vitamin D status) were associated with increased risks of LRTI in children in Bangladesh [13], India [14], and Turkey [15] and an increased risk of URTI in children in Canada [16], suggesting that vitamin D protects against URTI/LRTI. However, results from randomized controlled trials (RCTs) of vitamin D supplementation are variable [17–22]. A meta-analysis that combined individual data (adults and children) from 25 RCTs (n = 10 933) found that vitamin D supplementation reduced ARI risk among all participants (adjusted odds ratio: 0.88; 95% CI: 0.81-0.96) [23]. However, none of the studies in the meta-analysis involved maternal vitamin D supplementation, which is highly effective at improving neonatal and early infant vitamin D status in populations where maternal vitamin D deficiency is common [24]. Studies that included infants but were not included in the meta-analysis showed mixed results [25–27]. An RCT in New Zealand randomized healthy pregnant women from 27 weeks of gestational age, and their infants from birth to 6 months of age, to receive placebo, low-, or high-dose vitamin D_3_; compared with placebo, a lower proportion of children in the high-dose group made any healthcare visit for ARI up to 18 months of age compared with children in the placebo group but found no difference in the first 6 months of life [28].

It remains unknown whether maternal vitamin D supplementation reduces the risk of ARI during early infancy. In addition, it remains uncertain whether the risk of ARI due to specific respiratory viruses, such as RSV and influenza, is impacted by vitamin D supplementation. The Maternal Vitamin D Supplementation During Pregnancy and Lactation to Prevent Acute Respiratory Infections (MDARI) study aimed to determine if maternal vitamin D supplementation during pregnancy and lactation reduced the risk of microbiologically confirmed ARI in the first 6 months of life in infants born in Dhaka, Bangladesh.

## METHODS

### Study Design

MDARI (ClinicalTrials.gov identifier: NCT02388516; first posted March 17, 2015) was a prospective cohort study nested within a randomized, double-blind, placebo-controlled, dose-ranging parallel 5-treatment group trial of maternal vitamin D supplementation, the Maternal Vitamin D for Infant Growth (MDIG, ClinicalTrials.gov identifier: NCT01924013; first posted August 16, 2013) trial, which was conducted in Dhaka, Bangladesh. MDARI was designed to use rigorous active and passive surveillance to identify incident ARI in infants born to women enrolled in MDIG. Detailed methods for MDARI [29] and the protocol and results of the MDIG trial [24, 30] have been previously published. Recruitment and enrollment for MDARI took place at the Maternal and Child Health Training Institute (MCHTI), a public hospital in Dhaka, Bangladesh, or the homes of participants enrolled in MDIG.

MDIG participants (n = 1300) were randomly assigned to 1 of the 5 treatment groups. One group received 0 IU vitamin D per week during pregnancy and for 26 weeks post delivery (group A, “placebo”: 0; 0). Three groups received prenatal vitamin D supplementation at “low-dose” of 4200 IU per week (group B: 4200; 0), “mid-dose” of 16 800 IU per week (group C: 16 800; 0), or “high-dose” of 28 000 IU per week (group D: 28 000; 0), and then 0 IU per week for 26 weeks post delivery. A fifth group received high-dose prenatal vitamin D supplementation of 28 000 IU per week and the same dose until 26 weeks postpartum (group E: 28 000; 28 000). All doses, including placebo, were identical in appearance and taste and were delivered weekly to participants as described previously [29, 30]. Ingestion was directly observed by study personnel, whenever feasible. Participants, study staff, and investigators were blinded to the treatment group. MDARI protocol was approved by ethical review committees at the International Centre for Diarrhoeal Disease Research (icddr,b) (ERC protocol no. PR-14079) and The Hospital for Sick Children (REB no. 1000039072).

### Participants

All infants born to participants in MDIG were eligible for inclusion in MDARI. Pregnant women enrolled in MDIG were at least 18 years of age, generally healthy, and between 17 and 24 weeks of completed gestation based on a combination of last menstrual period and ultrasound. Enrollment into MDARI preferentially took place at the 30-week gestational age MDIG study visit; however, enrollment was permitted at prenatal visits after 30 weeks of gestational age, at delivery, and during postnatal visits prior to an infant reaching 6 months of age. MDARI enrollment started December 2, 2014, and ended March 3, 2016. All women provided written informed consent for participation in MDARI and were free to withdraw from MDARI without affecting their participation in MDIG. Withdrawal from MDIG resulted in automatic withdrawal from MDARI.

### Procedures

#### Surveillance

Active and passive surveillance for new, worsened, or persistent cases of clinical ARI was conducted from birth or enrollment (whichever was later) until 6 months of age. Active surveillance visits were conducted on a weekly basis and were performed by trained study personnel, usually at the participant’s household.

At each surveillance visit, a standardized infant physical examination was performed, and caregivers were asked to report symptoms/signs related to ARI (Supplementary Table S2). The presence of one or more ARI-related symptoms/signs triggered a telephone call to a study physician who reviewed URTI and/or LRTI criteria (Table 1) to determine if nasal swab collection was indicated.

**Table 1.** Study Outcome Definitions

ARI Type	Case Definition
Clinical URTI	A new-onset illness consisting of at least 2 of the following clinical criteria at any time during a surveillance week: • Caregiver-reported cough; • Caregiver-reported rhinorrhea; • Caregiver-reported nasal congestion; • Measured temperature ≥ 37.5°C (axillary) confirmed with the second measurement.
Clinical LRTI	Caregiver-reported cough AND/OR difficulty breathing during a surveillance week AND Observed elevated respiratory rate (60 breaths per min or greater for infant up to 59 d of age, or 50 breaths per min or greater for infant 60 d of age or older) and/or lower chest wall in-drawing; OR Hospitalization with physician diagnosis of pneumonia or bronchiolitis (referred to as “Hospitalized LRTI”).
Microbiologically confirmed URTI	Clinical URTI (as defined above) accompanied by a positive test for at least one of influenza A; influenza B; respiratory syncytial virus; parainfluenza 1, 2, and 3; adenovirus; or human metapneumovirus.
Microbiologically confirmed LRTI	Clinical LRTI (as defined above) accompanied by a positive test for at least one of influenza A; influenza B; respiratory syncytial virus; parainfluenza 1, 2, and 3; adenovirus; or human metapneumovirus.

Abbreviations: ARI, acute respiratory infection; URTI, upper respiratory tract infection; LRTI, lower respiratory tract infection.

To identify ARI cases between scheduled visits, caregivers were encouraged to contact the study physician directly by telephone if their infant developed signs of ARI (Supplementary Table S2) or had been hospitalized. An incentive of approximately $0.25 USD cell phone credit was provided to caregivers who reported an ARI-related symptom. If criteria were met for swab collection, additional credit was given for a total of approximately $2.50 USD. Passively reported ARI features were verified by trained study personnel at an in-home or clinic visit, and a nasal swab was collected if ARI criteria were confirmed. Study physicians also reported ARI signs/symptoms that were detected during any participant encounter, which could trigger nasal swab collection.

#### Nasal Swab Collection and Microbiologic Testing

Nasal swab collection procedures and molecular testing protocols were previously published [29]. Among infants who met clinical ARI criteria, swab collection was only performed if a swab had not been collected in the past 7 days, and there was at least one study visit in the preceding surveillance week where clinical ARI criteria were not met; or relative to the preceding surveillance week, the ARI had clinically worsened (ie, URTI worsened to LRTI); or at least 4 study weeks had passed since the last swab was collected. If criteria were satisfied, a swab was collected, transported to the Virology Lab at icddr,b, and processed as previously described [29].

Individual real-time 1-step reverse transcriptase polymerase chain reaction (qRT-PCR) assays were performed on extracted nucleic acids using primer and probe sequences as recommended by the US Centers for Disease Control and Prevention (CDC) to detect influenza A and B [29]. Individual qRT-PCR assays were also performed for adenovirus, human metapneumovirus, human parainfluenza 1–3, and RSV using CDC-recommended primer and probe sequences [29].

#### Outcomes

The primary outcome was microbiologically confirmed viral-associated ARI (Table 1). Main secondary outcomes included ARI with microbiologically confirmed influenza A or B, ARI with microbiologically confirmed RSV, and clinical ARI, URTI, and/or LRTI (HLRTI) (Table 1). LRTIs were further subtyped as “hospitalized LRTI” if the infant was admitted at any time during the ARI episode.

#### Analysis Plan

The analysis was conducted as a complete-case intention-to-treat analysis. Incidence rates (IRs) with 95% CI of microbiologically confirmed ARI were estimated using Cox proportional hazards model with the Andersen-Gill extension. Jackknife estimators of standard errors were used to account for nonindependence of recurrent events (ie, repeated ARI episodes within the same infant). All analyses were conducted using age (in weeks) as the time scale. For women who were enrolled prenatally or at delivery, follow-up was initiated at birth. For infants who were enrolled post delivery, follow-up was initiated when consent was obtained. Infants were censored if lost to follow-up or otherwise at 26 weeks of age.

Infants contributed a person-week to the analysis only if they were considered to be at-risk for an incident ARI during that week. To avoid misattributing common newborn respiratory conditions (eg, transient tachypnea) to ARI, the first week of life was not considered at risk. Therefore, infants contributed time at-risk starting from week 2 of life, up until clinical and/or microbiological confirmation of ARI. After an ARI episode, infants could be considered at risk again (and contribute person-time) if the infant had a documented week in which they no longer met the clinical ARI criteria or more than 4 study weeks had elapsed since the onset of the previous ARI. In sub-analyses, URTI and LRTI were considered separate outcomes, such that an infant with a URTI in 1 week would be at risk for LRTI in the current or next study week. However, an infant with LRTI in one study week was not considered at risk for URTI in the current or following week (ie, URTI features were assumed to be due to the same, and perhaps improving, illness episode as the preceding LRTI).

The study was designed with 80% power to detect a 26% reduction (hazard ratio [HR] = 0.74) in the rate of microbiologically confirmed ARI among infants in the highest-dose vitamin D supplementation group including postpartum supplementation (group E) compared with the placebo group (group A), assuming an IR of microbiologically confirmed ARI in group A of 3 cases per child-year and that up to 10% of MDIG participants may be lost to follow-up or not consent to MDARI.

A gatekeeper approach for the primary analysis was planned, whereby formal comparisons of each lower-dose group (vs placebo) would proceed only if the primary comparison of group E vs A was statistically significant (*P* < .05). In exploratory secondary analyses, we compared an aggregated high-dose vitamin D group (group C, 16 800; 0, or group D, 28 000; 0) to a combined low-dose or no vitamin D group (group B, 4200; 0, or group A placebo, 0; 0); in accordance with the original statistical plan, this approach aimed to optimize power for detecting small between-group differences for which we were not powered in the primary analysis. In further post hoc secondary analyses, a pairwise comparison was made between groups E and D to identify the impact of continuing vitamin D supplementation post delivery on the incidence of microbiologically confirmed ARI and we compared the IR for ARI with RSV, or with influenza A or B, across supplementation groups. Analyses of secondary outcomes were conducted as described for primary outcomes or as outlined in supplementary tables.

Numerous sensitivity analyses were conducted to test the robustness of the primary analysis, including variations to the at-risk definition, unit of analysis, case definition, surveillance and swab parameters, and using the prevalence of the outcome rather than rate.

## RESULTS

**Figure 1.**
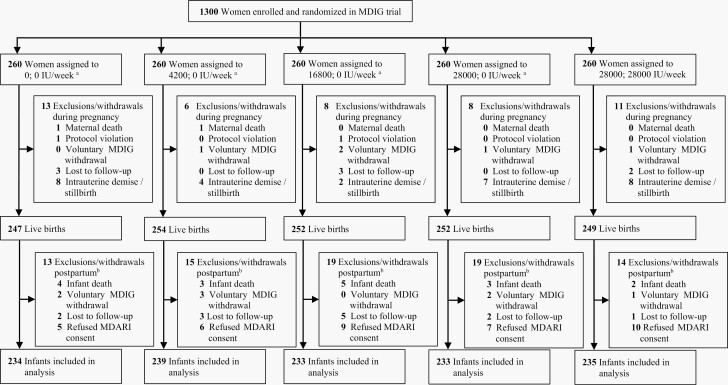
CONSORT flow diagram of infants included in MDARI. ^a^Prenatal; postpartum vitamin D dose. ^b^Exclusion/withdrawals in the postpartum period after enrollment in MDIG but prior to enrollment in MDARI. Abbreviations: MDIG, Maternal Vitamin D for Infant Growth; MDARI, Maternal Vitamin D Supplementation During Pregnancy and Lactation to Prevent Acute Respiratory Infections.

MDARI included 1174 (90%) of the women enrolled in MDIG and the number of infants in each of the treatment arms ranged from 233 to 239 (Figure 1). Sixty-four percent of women were enrolled in MDARI prenatally and cumulatively 71% and 77% of infants were enrolled by 1 week and 4 weeks of age, respectively. The distributions of maternal characteristics and maternal baseline serum 25(OH)D concentration were similar between MDARI and MDIG participants (Supplementary Table S3) and across supplementation groups in MDARI (Table 2). Overall adherence to scheduled doses was high and similar across all supplementation groups (Supplementary Table S4). Strong effects of vitamin D dose on maternal serum 25(OH)D concentration at or near delivery, and infant vitamin D status at 6 months of age, were observed (Supplementary Table S5) as in MDIG [24]. There were no differences across supplementation arms in MDARI for anthropometric measures at or just after birth (Supplementary Table S6).

**Table 2.** Maternal Characteristics at Enrollment (Second Trimester of Pregnancy) for Infants Enrolled in MDARI, by Supplementation Group

	Prenatal; Postpartum Vitamin D Dose (IU/wk)				
	0; 0	4200; 0	16 800; 0	28 000; 0	28 000; 28 000
Participants, N	234	239	233	233	235
Maternal age (y), median (minimum, maximum)	23 (18, 38)	23 (18, 40)	22 (18, 35)	22 (18, 38)	23 (18, 38)
Gestational age (wk) at enrollment if enrolled in prenatal period, median (minimum, maximum)	20.4 (17, 24)	20.3 (17, 24)	20.3 (17, 24)	20.4 (17, 24)	20.1 (17, 24)
Age at enrollment if enrolled in postnatal period (wk), median (minimum, maximum)^a^	9.0 (0, 23)	6.0 (0, 26)	8.1 (0, 21)	8.1 (0, 23)	8.0 (0, 26)
Marital status, n (%)^b^					
Married	232 (99.1)	239 (100.0)	233 (100.0)	231 (100.0)	235 (100.0)
Not married	2 (0.9)	0 (0.0)	0 (0.0)	0 (0.0)	0 (0.0)
Level of education, n (%)					
No schooling	9 (3.8)	10 (4.2)	10 (4.3)	10 (4.3)	8 (3.4)
Primary incomplete	52 (22.2)	45 (18.8)	49 (21.0)	50 (21.5)	54 (23.0)
Primary complete	33 (14.1)	36 (15.1)	23 (9.9)	35 (15.0)	38 (16.2)
Secondary incomplete	92 (39.3)	83 (34.7)	103 (44.2)	87 (37.3)	84 (35.7)
Secondary complete or higher	48 (20.5)	65 (27.2)	48 (20.6)	51 (21.9)	51 (21.7)
Primary occupation, n (%)^b^					
Homemaker	218 (93.2)	223 (93.3)	219 (94.0)	215 (93.1)	221 (94.0)
Other	16 (6.8)	16 (6.7)	14 (6.0)	16 (6.9)	14 (6.0)
Asset index quintiles, n (%)^c^					
1 (lowest)	50 (21.4)	55 (23.1)	33 (14.2)	55 (23.9)	44 (18.7)
2	43 (18.4)	48 (20.2)	56 (24.1)	38 (16.5)	40 (17.0)
3	55 (23.5)	39 (16.4)	49 (21.1)	48 (20.9)	50 (21.3)
4	42 (17.9)	39 (16.4)	51 (22.0)	49 (21.3)	50 (21.3)
5 (highest)	44 (18.8)	57 (23.9)	43 (18.5)	40 (17.4)	51 (21.7)
Month of enrollment, n (%)					
March-May	86 (36.8)	80 (33.5)	90 (38.6)	83 (35.6)	79 (33.6)
June-August	76 (32.5)	74 (31.0)	69 (29.6)	81 (34.8)	74 (31.5)
September-November	37 (15.8)	47 (19.7)	40 (17.2)	36 (15.5)	46 (19.6)
December-February	35 (15.0)	38 (15.9)	34 (14.6)	33 (14.2)	36 (15.3)
Maternal serum 25(OH)D concentration (nmol/L) at enrollment, mean ± SD^d^	27.2 ± 13.9	27.6 ± 14.4	28.4 ± 14.0	27.4 ± 15.1	26.4 ± 13.3
Venous umbilical cord 25(OH)D concentration <30 nmol/L at birth, n (%)	96 (98)	22 (22)	0 (0)	0 (0)	0 (0)

Abbreviation: MDARI, Maternal Vitamin D Supplementation During Pregnancy and Lactation to Prevent Acute Respiratory Infections.

^a^N_0; 0_ = 73, N_4200; 0_ = 87, N_16 800; 0_ = 78, N_28 000; 0_ = 84, N_28 000; 28 000_ = 88.

^b^N_0; 0_ = 234, N4_200; 0_ = 239, N_16 800; 0_ = 233, N_28 000; 0_ = 231, N_28 000; 28 000_ = 235.

^c^N_0; 0_ = 234, N_4200; 0_ = 238, N_16 800; 0_ = 232, N_28 000; 0_ = 230, N_28 000; 28 000_ = 235.

^d^N_0; 0_ = 233, N_4200; 0_ = 238, N_16 800; 0_ = 232, N_28 000; 0_ = 232, N_28 000; 28 000_ = 234.

Ninety percent of infants were born at term (≥37 weeks to <42 weeks), more than half were born by cesarean section, fewer than 15% were born outside of a hospital or clinic, and there were no differences in these characteristics across supplementation arms (Supplementary Table S6). The median duration of infant follow-up was 26 weeks across all supplementation groups (Supplementary Table S7). Ninety percent of infants had at least one incident clinical ARI episode. The numbers and types of ARIs identified across groups, by surveillance type, are presented in Supplementary Tables S7 and S8. The first microbiologically confirmed ARI was on December 4, 2014, and the last on August 10, 2016. A total of 3776 swabs were collected. About 90% of infants had at least one swab collected, and the median number of swabs per infant was 3 (interquartile range [IQR] 2-5). 

**Figure 2.**
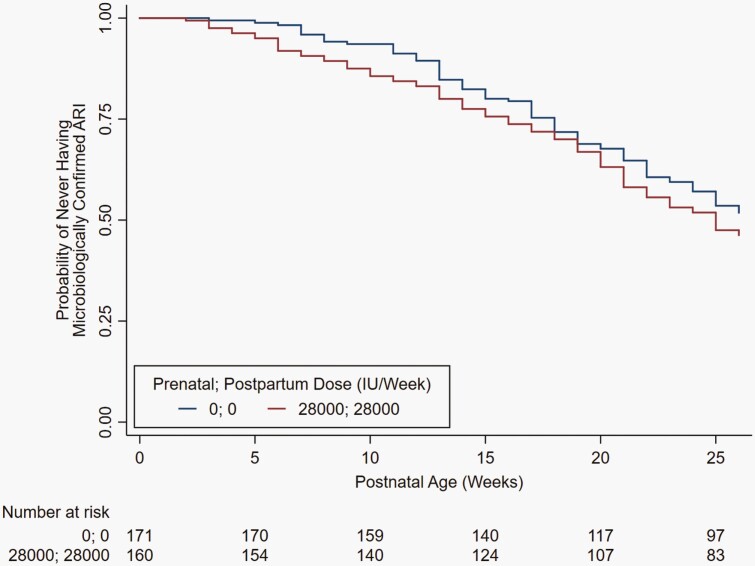
Kaplan-Meier curve for not experiencing a first microbiologically confirmed ARI in the first 26 weeks of age in the high-dose maternal prenatal and postpartum vitamin D supplementation (28 000 IU/week) group vs placebo group among infants enrolled from birth (n = 331). Abbreviation: ARI, acute respiratory infection.

The rates of microbiologically confirmed ARI in the high-dose prenatal and postnatal supplementation group E (IR = 1.21 cases/6 person-months) compared with group A (IR = 1.07 cases/6 person-months) were not significantly different (HR = 1.12, 95% CI: 0.90-1.40), and median time to first episode of ARI was comparable between the 2 groups (17 weeks) (Table 3). No differences were observed in the incidence of microbiologically confirmed or clinical ARI, URTI, LRTI, or hospitalized LRTI between group E and placebo group A. Microbiologically confirmed ARI-free survival among infants followed from birth was comparable between groups E and A (Figure 2) and across all groups (Supplementary Figure S1). Recurrent LRTI was relatively rare (Supplementary Table S9). The most common virus associated with URTI was parainfluenza (Supplementary Tables S10 and S11), and the virus most often identified in LRTI was RSV (Supplementary Tables S10 and S11).

**Table 3.** Effect of High-Dose Maternal Prenatal and Postpartum Vitamin D Supplementation (28 000 IU/wk) vs Placebo (0 IU/wk) on Incidence of Acute Respiratory Infections in Infants From 0 to 6 Months of Age

	0; 0 (n = 234)				28 000; 28 000 (n = 235)				HR (95% CI)	*P*-value^a^
	Number of Incident Episodes	Person-Time at Risk (wk)	Incidence Rate (Per 6 Person-Months^c^)	Time to First Episode (wk), Median (IQR)	Number of Incident Episodes	Person-Time at Risk (wk)	Incidence Rate (Per 6 Person-Months^c^)	Time to First Episode (wk), Median (IQR)		
Microbiologically confirmed ARI										
ARI	154	3728	1.07	17 (13, 22)	171	3679	1.21	17 (10, 21)	1.12 (0.90, 1.40)	.315
URTI	154	3720	1.08	17 (13, 22)	171	3676	1.21	17 (10, 21)	1.12 (0.90, 1.40)	.317
LRTI^b^	22	4693	0.12	15 (13, 23)	19	4604	0.11	13 (9, 18)	0.87 (0.46, 1.66)	.675
HLRTI	10	4736	0.05	22 (15, 25)	8	4634	0.04	14 (9.5, 19)	0.82 (0.31, 2.15)	.682
ARI with RSV	36	3728	0.25	20 (13, 23)	36	3679	0.25	19.5 (9.5, 23)	1.01 (0.62, 1.63)	.980
ARI with influenza A/B	20	3728	0.14	21 (15, 24)	26	3679	0.18	19 (17, 23)	1.30 (0.71, 2.40)	.393
Clinical ARI										
ARI	724	3728	5.05	9 (5, 16)	704	3679	4.98	10 (5, 17)	0.98 (0.85, 1.13)	.774
URTI	722	3719	5.05	9 (5, 16)	703	3677	4.97	10 (5, 17)	0.98 (0.85, 1.13)	.775
LRTI^b^	60	4692	0.33	13 (8, 21)	40	4604	0.23	12 (6, 18)	0.67 (0.41, 1.12)	.128
HLRTI	17	4735	0.09	19 (11, 24)	11	4634	0.06	13 (8, 20)	0.66 (0.30, 1.46)	.305

Abbreviations: ARI, acute respiratory infection; CI, confidence interval; HR, hazard ratio; LRTI, lower respiratory tract infection; RSV, respiratory syncytial virus; URTI, upper respiratory tract infection.

^a^ *P*-values from a cox proportional hazards model. Jackknife estimation of standard errors was used to account for repeated events within the same infant.

^b^Nonhospitalized and hospitalized cases of LRTI.

^c^Analyses conducted using 26 wk.

No differences in rates of microbiologically confirmed or clinical ARI of any type were observed when comparing no/low vs mid-/high-dose groups (Supplementary Table S12), and conclusions were the same when considering prevalence as the outcome (Supplementary Table S13). There were no differences between groups D and E (Supplementary Table S14). Overall, ARI IRs (Table 4) and cumulative incidences (Supplementary Table S15) were similar across all supplementation groups. No differences were observed in IR for ARI with RSV, or with influenza A or B, across supplementation groups (Table 4). Frequencies of signs/symptoms among incident ARI cases were similar across supplementation groups (Supplementary Table S16). Primary findings were similar after adjusting or stratifying on key risk factors (Supplementary Tables S17 and S18), and all sensitivity analyses supported the conclusion that maternal vitamin D supplementation had no effect on the risk of ARI in infancy (Supplementary Tables S19–S22).

**Table 4.** Incidence Rates of Microbiologically Confirmed ARI and Clinical ARI Among Infants From 0 to 6 Months of Age, by Supplementation Group

		Prenatal; Postpartum Vitamin D Dose (IU/wk)				
		0; 0	4200; 0	16 800; 0	28 000; 0	28 000; 28 000
		(n = 234)	(n = 239)	(n = 233)	(n = 233)	(n = 235)
Microbiologically confirmed						
ARI	Number of incident episodes	154	155	174	166	171
	Person-time at risk, wk	3728	3736	3629	3526	3679
	Incidence rate (95% CI), per 6 person-months^a^	1.07 (0.92, 1.26)	1.08 (0.93, 1.25)	1.25 (1.08, 1.44)	1.22 (1.04, 1.44)	1.21 (1.03, 1.41)
URTI	Number of incident episodes	154	155	174	163	171
	Person-time at risk, wk	3720	3730	3625	3522	3676
	Incidence rate (95% CI), per 6 person-months^a^	1.08 (0.92, 1.26)	1.08 (0.93, 1.25)	1.25 (1.08, 1.44)	1.20 (1.02, 1.42)	1.21 (1.04, 1.41)
LRTI^b^	Number of incident episodes	22	17	31	26	19
	Person-time at risk, wk	4693	4716	4633	4551	4604
	Incidence rate (95% CI), per 6 person-months^a^	0.12 (0.08, 0.19)	0.09 (0.06, 0.15)	0.17 (0.12, 0.25)	0.15 (0.10, 0.22)	0.11 (0.07, 0.17)
HLRTI	Number of incident episodes	10	8	18	11	8
	Person-time at risk, wk	4736	4755	4678	4590	4634
	Incidence rate (95% CI), per 6 person-months^a^	0.05 (0.03, 0.10)	0.04 (0.02, 0.09)	0.10 (0.06, 0.16)	0.06 (0.03, 0.11)	0.04 (0.02, 0.09)
ARI with RSV	Number of incident episodes	36	30	35	36	36
	Person-time at risk, wk	3728	3736	3629	3526	3679
	Incidence rate (95% CI), per 6 person-months^a^	0.25 (0.18, 0.35)	0.21 (0.14, 0.30)	0.25 (0.18, 0.36)	0.27 (0.19, 0.38)	0.25 (0.18, 0.36)
ARI with influenza A/B	Number of incident episodes	20	22	15	27	26
	Person-time at risk, wk	3728	3736	3629	3526	3679
	Incidence rate (95% CI), per 6 person-months^a^	0.14 (0.09, 0.22)	0.15 (0.10, 0.24)	0.11 (0.06, 0.18)	0.20 (0.13, 0.30)	0.18 (0.12, 0.27)
Clinical criteria						
ARI	Number of incident episodes	724	730	768	761	704
	Person-time at risk, wk	3728	3736	3628	3525	3679
	Incidence rate (95% CI), per 6 person-months^a^	5.05 (4.59, 5.55)	5.08 (4.62, 5.58)	5.50 (5.01, 6.04)	5.61 (5.12, 6.15)	4.98 (4.51, 5.49)
URTI	Number of incident episodes	722	727	763	758	703
	Person-time at risk, wk	3719	3730	3624	3521	3677
	Incidence rate (95% CI), per 6 person-months^a^	5.05 (4.59, 5.55)	5.07 (4.61, 5.57)	5.47 (4.99, 6.01)	5.60 (5.11, 6.13)	4.97 (4.51, 5.48)
LRTI^b^	Number of incident episodes	60	51	70	57	40
	Person-time at risk, wk	4692	4716	4631	4550	4604
	Incidence rate (95% CI), per 6 person-months^a^	0.33 (0.24, 0.46)	0.28 (0.21, 0.37)	0.39 (0.29, 0.52)	0.33 (0.24, 0.44)	0.23 (0.15, 0.33)
HLRTI	Number of incident episodes	17	12	27	19	11
	Person-time at risk, wk	4735	4755	4676	4589	4634
	Incidence rate (95% CI), per 6 person-months^a^	0.09 (0.06, 0.15)	0.07 (0.04, 0.12)	0.15 (0.10, 0.22)	0.11 (0.07, 0.17)	0.06 (0.03, 0.11)

Abbreviations: ARI, acute respiratory infection; CI, confidence interval; LRTI, lower respiratory tract infection; RSV, respiratory syncytial virus; URTI, upper respiratory tract infection.

^a^Jackknife estimation of standard errors was used to account for repeated events within the same infant.

^b^Nonhospitalized and hospitalized cases of LRTI.

## Discussion

MDARI leveraged the parallel, 5-arm, randomized design of the MDIG trial to determine whether vitamin D supplementation during pregnancy and lactation could prevent early infant ARI in Dhaka, Bangladesh, a setting where both the prevalence of vitamin D deficiency and the burden of ARI are high. Vitamin D did not impact the IR of microbiologically confirmed ARI or clinical ARI, URTI, LRTI, or ARI from RSV or influenza in early infancy. These findings contrast with a recent meta-analysis [23] of the effect of vitamin D supplementation on ARI in studies involving primarily adults and older children, which concluded that vitamin D supplementation offered protection, particularly among individuals with baseline 25(OH)D concentrations less than 25 nmol/L and in those who received daily or weekly dosing of vitamin D (adjusted odds ratio [aOR]: 0.30, 95% CI: 0.17-0.53). All MDARI participants received weekly supplementation and many pregnant women had baseline 25(OH)D concentrations below 30 nmol/L, and yet vitamin D did not influence the risk of ARI in their offspring. Only 4 studies included in the meta-analysis included participants under 1 year of age and a subgroup analysis of these infants revealed no reduction in the proportion of infants who experienced at least one ARI (aOR: 0.94, 95% CI: 0.83-1.06). One other RCT of maternal vitamin D supplementation during pregnancy conducted in a high-income country found that vitamin D did not reduce primary care visits for ARI in infants under 6 months of age in New Zealand [28].

Few previous trials that have explored the effect of vitamin D on the risk of ARI have included molecular testing; therefore, data on vitamin D’s potential to reduce the incidence of infection due to specific viruses are scant. While MDARI was not powered to detect differences in virus-specific outcomes, maternal vitamin D supplementation did not influence the IR of ARI with influenza A or B, or RSV, or time to first episode of infection from these viruses. Prior to MDARI, there were no published data on the effect of vitamin D on the risk of RSV, and data on the effect of vitamin D supplementation on the risk of influenza are mixed. A trial conducted in Japanese children that administered 1200 IU vitamin D daily reported a reduction in influenza A (relative risk: 0.58; 95% CI: 0.34–0.99) [31]. Similarly, a trial in Vietnam showed a reduction in the incidence of influenza A and duration of symptoms in infants who received high-dose (1200 IU daily) compared with low-dose vitamin D (400 IU daily) [27]. However, other studies found no evidence of decreased risk of influenza in children and adolescents [17, 32]. Given the importance of influenza and RSV to child health, the uncertainty surrounding vitamin D’s effect on influenza and RSV warrants investigation in adequately powered trials.

Variability in the operational definition of ARI can affect reported estimates for the IR of ARI [33]. The use of microbiologically confirmed ARI as the study’s primary outcome, and the viruses selected for molecular testing, increased the stringency of the outcome definition compared with clinical criteria alone. Some viruses (rhinovirus, enterovirus, and seasonal coronaviruses) were excluded from the PCR panel due to evidence that they may be found in asymptomatic children and their inclusion may blunt differences between groups [34]. Thus, a limitation of this study is that an ARI caused by a virus not included would not have met the primary outcome definition; nonetheless, our inferences were similar in analyses that considered all clinical ARI episodes. A further limitation is that this study was underpowered for LRTI and HLRTI. A major strength of this study was the active and passive surveillance system used to identify and confirm potential clinical ARI events prior to microbiologic testing. Notably, the primary outcome event rate in this study was very similar to the predicted rate used in the sample size calculation. This study was conducted before the first documented case of infection with severe acute respiratory syndrome coronavirus 2 (SARS-CoV-2) in Bangladesh and thus no inferences about maternal vitamin D supplementation and infection with this virus in early infancy can be made.

## Conclusions

The MDARI findings support the World Health Organization (WHO) position [35] that vitamin D supplementation should not be routinely recommended during pregnancy to improve maternal and perinatal outcomes and suggest that future guidelines could be extended to acknowledge that prenatal vitamin D supplementation should not be recommended for ARI prevention during infancy, even in locations where vitamin D deficiency in pregnancy is common.

## Supplementary Material

piab032_suppl_Supplementary_Material

## Notes

***Acknowledgments***. We thank the trial participants and their families; the staff of the International Centre for Diarrhoeal Disease Research, Bangladesh (icddr,b), who implemented the trial and collected data; the staff of the Maternal and Child Health Training Institute and Kazi Moksedur Rahman (executive director, Shimantik) for their collaboration; the Ministry of Health and Family Welfare of Bangladesh for its approval to conduct the MDIG trial; present and former staff at the Centre for Global Child Health at The Hospital for Sick Children (Toronto), including Jason Black, Nadine Francis, Jill Korsiak, Huma Qamar, Shruti Mallya, and Vivian Tam; and the staff at the Bill and Melinda Gates Foundation.

***Authors contributions***. S. K. M. and D. E. R. conceived the study and led the protocol design. S. K. M. wrote the first draft of the manuscript. A. A. M. and M. Z. R. lead the protocol implementation and laboratory analyses, respectively, in Bangladesh. L. G. P., J. S., T. A., M. C. D., J. B. G., M. M. I., T. K., F. K. K., M. M., E. P., M. S., S. S. S., M. K. S., and S. Z. contributed to study design, analysis, and/or implementation. All authors read and approved the final manuscript. S. K. M. and D. E. R. had full access to all the data in the study and take responsibility for the integrity of the data and the accuracy of the data analysis.

***Role of the funding source***. The funders of the study had no role in study design, data collection, analysis, interpretation, or writing of the report.

***Previous presentation*:** This work was previously presented in part as an abstract (#1244) at the 2017 American Society of Tropical Health and Hygiene 66th Annual Meeting, Baltimore, MD.

***Financial support***. This work was supported by the Bill and Melinda Gates Foundation (grant number OPP1066764).

***Potential conflicts of interest***. All authors: No reported conflicts.

Data Availability:

De-identified individual participant data that underlie the results reported in this article (text, tables, figures, and appendices) and the study protocol will be available immediately after publication and ending 5 years following article publication. These data and documents will be made available to investigators whose secondary data analysis study protocol has been approved by an independent research ethics board. Proposals should be directed to daniel.roth@sickkids.ca; to gain access, data requestors will need to sign a data access agreement.
