# Supplementary material for: Effects of Maternal Vitamin D Supplementation During Pregnancy and Lactation on Infant Acute Respiratory Infections: Follow-up of a Randomized Trial in Bangladesh
Source: J Pediatric Infect Dis Soc. 2021 Jul 2;10(9):901–9. doi: 10.1093/jpids/piab032 (PMC8557369; doi:10.1093/jpids/piab032)

**Supplemental Material**

**Title**

Maternal Vitamin D Supplementation during Pregnancy and Lactation to Prevent Acute Respiratory Infections in Infancy

Shaun K. Morris, MD^1,2,3,4^, Lisa G. Pell, PhD^1^, Mohammed Ziaur Rahman, PhD^5^, Abdullah Al Mahmud, MMSc^6^, Joy Shi, MSc^1,7^, Tahmeed Ahmed, PhD^6*^, Michelle C. Dimitris, PhD^1*^, Jonathan B. Gubbay, MBBS^2,8*^, M. Munirul Islam, PhD^6*^, Tahmid Kashem, MPH^9*^, Farhana K. Keya, MBBS^6*^, Minhazul Mohsin, MBBS^6*^, Eleanor Pullenayegum, PhD^3,10*^, Michelle Science, MD^2,4*^, Shaila S. Shanta, MBBS^6*^, Mariya K. Sumiya, MS^5*^, Stanley Zlotkin, MD^1,2,3*^, Daniel E. Roth, MD^1,2,3^

^1^ Centre for Global Child Health, The Hospital for Sick Children, Toronto, Ontario, Canada

^2^ Department of Pediatrics, University of Toronto and The Hospital for Sick Children, Toronto, Ontario, Canada

^3^ Child Health Evaluative Sciences, The Hospital for Sick Children, Toronto, Ontario, Canada

^4^ Division of Infectious Diseases, The Hospital for Sick Children, Toronto, Ontario, Canada

^5^ Infectious Diseases Division, International Centre for Diarrhoeal Disease Research, Bangladesh, Dhaka, Bangladesh

^6^ Nutrition and Clinical Services Division, International Centre for Diarrhoeal Disease Research, Bangladesh, Dhaka, Bangladesh

^7^ Department of Epidemiology, Harvard University T H Chan School of Public Health, Boston, Massachusetts, USA

^8^ Public Health Ontario, Toronto, Ontario, Canada

^9^ Primary and Community Health Branch, Ministry of Health, Edmonton, Alberta, Canada

^10^ Division of Biostatistics, Dalla Lana School of Public Health, University of Toronto, Toronto, Ontario, Canada

* Authors in positions 6 through 17 in alphabetical order

**Contents**

[Tables](#_Toc47956186)

[Table S1. Participant inclusion and exclusion criteria 4](#_Toc47956187)

[Table S2. Surveillance for signs and symptoms 5](#_Toc47956188)

[Table S3. Comparison of mothers enrolled in the MDIG trial versus mothers whose infant is enrolled in the MDARI study 6](#_Toc47956189)

[Table S4. Supplementation duration and adherence among mothers, by supplementation group 7](#_Toc47956190)

[Table S5. Maternal and infant 25-hydroxyvitamin D concentrations among mother-infant pairs enrolled in the MDARI study, overall and by supplementation group 8](#_Toc47956191)

[Table S6. Delivery characteristics and pregnancy outcomes, by supplementation group 9](#_Toc47956192)

[Table S7. ARI data collection via active and passive surveillance systems, by supplementation group 11](#_Toc47956193)

[Table S8. ARI reported by CHW, caregiver, and study physician, by supplementation group 13](#_Toc47956194)

Table S9. Number of episodes of LRTI per infant from 0 to 6 months of age, by supplementation group……………………………………….14

[Table S10. Respiratory viruses identified in episodes of microbiologically confirmed ARI by ARI type 15](#_Toc47956195)

[Table S11. Respiratory viruses identified in episodes of clinical ARI by ARI type 16](#_Toc47956196)

[Table S12. Effect of mid- or high-dose prenatal vitamin D supplementation (16,800 or 28,000 IU) versus placebo (0 IU/week) or low-dose (4,200 IU/week) vitamin D supplementation on incidence of acute respiratory infections up to 6 months of age 17](#_Toc47956197)

[Table S13. Period prevalence of microbiologically confirmed ARI and clinical ARI among infants from 0 to 6 months of age, by supplementation group 18](#_Toc47956198)

[Table S14. Effect of 28,000 IU vitamin D per week versus placebo in the postpartum period on incidence of infant acute respiratory infections among infants born to women administered 28,000 IU/week vitamin D in the prenatal period 19](#_Toc47956199)

[Table S15. Cumulative incidence of at least one episode of ARI, by supplementation group 20](#_Toc47956200)

[Table S16. Caregiver-reported and study worker-observed clinical signs and identified hospitalization among incident ARI cases, by supplementation group 21](#_Toc47956201)

[Table S17. Unadjusted and adjusted analyses of associations between participant or household characteristics and incidence of microbiologically confirmed acute respiratory infections in infants from 0 to 6 months of age 22](#_Toc47956202)

[Table S18. Effect of maternal 28,000 IU/week vitamin D supplementation in prenatal and postpartum periods versus placebo, on the incidence of microbiologically confirmed acute respiratory infections in infants from 0 to 6 months of age, by sex, season, month and breastfeeding status 23](#_Toc47956203)

[Table S19. Sensitivity analyses of the effect of high-dose maternal prenatal and postpartum vitamin D supplementation (28,000 IU/week) versus placebo on incidence of microbiologically confirmed acute respiratory infections in infants from 0 to 6 months of age 24](#_Toc47956204)

[Table S20. Effect of high-dose maternal prenatal vitamin D supplementation (28,000 IU/week) with or without postpartum vitamin D (0 IU/week or 28,000 IU/week) versus placebo on incidence of microbiologically confirmed acute respiratory infections in infants from 0 to 6 months of age 27](#_Toc47956205)

[Table S21. Effect of maternal prenatal vitamin D supplementation of either 16,800 or 28,000 IU/week (with or without 28,000 IU/week in the postpartum period), versus placebo, on incidence of microbiologically confirmed acute respiratory infections in infants from 0 to 6 months of age 28](#_Toc47956206)

[Table S22. Effect of any dose of prenatal or postpartum maternal vitamin D supplementation versus placebo on incidence of microbiologically confirmed acute respiratory infections in infants from 0 to 6 months of age 29](#_Toc47956207)

[Figures](#_Toc47956208)

[Figure S1. Kaplan-Meier curve for not experiencing a first microbiologically confirmed ARI in the first 26 weeks of age among infants enrolled from birth (n = 833), by supplementation group 30](#_Toc47956209)

**Tables**

**Table S1. Participant inclusion and exclusion criteria**

| **Inclusion criteria** | **Exclusion criteria** |
| --- | --- |
| Age 18 years and above*  At 30 weeks of gestation up to 6 months postpartum (based on recalled LMP and/or ultrasound)^†^  *Rules for integrating information from recalled LMP and ultrasound:*  • If there is a difference of >10 days between gestational age dated using the LMP and second trimester ultrasound, the estimated date of delivery will be adjusted as per the second trimester ultrasound (SOGC guidelines); otherwise (i.e., if the difference is ≤10 days), the GA date based on LMP will be used • If there is more than one ultrasound, GA estimation should be based on the earliest of the ultrasounds for which a written report is available. If the earliest ultrasound was performed in the 1st trimester, and there is a difference of >5 days between gestational age dated using the LMP and 1st trimester ultrasound, the estimated date of delivery will be adjusted as per the 1st trimester ultrasound (SOGC guidelines); otherwise (i.e., if the difference is ≤5 days), the GA date based on LMP will be used  Intends to reside in the trial catchment area (including Hazaribag, Azimpur, Lalbag, and Kamrangirchar) for at least 18 months following enrolment into the MDIG trial*  Provides written informed consent*^†^ | History of any medical condition or medications that may predispose to vitamin D sensitivity, altered vitamin D metabolism, and/or hypercalcemia, including active tuberculosis or current therapy for tuberculosis, sarcoidosis, history of renal/ureteral stones, parathyroid disease, renal or liver failure, or current use of anti-convulsants*  High-risk pregnancy based on one or more of the following findings by point-of-care testing: severe anemia (hemoglobin <70 g/L assessed by Hemocue), moderate-severe proteinuria (≥300 mg/dl (3+ or 4+) based on urine dipstick), and hypertension (≥1 systolic blood pressure reading ≥140 mm Hg and/or ≥1 diastolic blood pressure reading ≥90 mm Hg in repeat measurements taken at least one minute apart)*  High-risk pregnancy based on one or more of the following findings by maternal history and/or ultrasound: multiple gestation, major congenital anomaly, and severe oligohydramnios*  Unwillingness to stop taking non-study vitamin D or calcium supplements or multivitamins containing calcium and/or vitamin D*  Currently prescribed vitamin D supplements as part of a physician's treatment plan for vitamin D deficiency*  Enrolment in the MDIG trial during a previous pregnancy*  Withdrawal from the MDIG trial^†^ |
| * denotes inclusion or exclusion criteria of the MDIG trial  ^†^ denotes additional inclusion or exclusion criteria specific to MDARI  LMP, last menstrual period; GA, gestational age; MDIG, Maternal vitamin D for Infant Growth. | |

**Table S2. Surveillance for signs and symptoms**

|  | **Active surveillance for clinical ARI** | **Passive surveillance for clinical ARI** |
| --- | --- | --- |
| Signs and symptoms | Caregiver-reported cough, rhinorrhea (i.e., runny nose), nasal congestion (i.e., stuffy nose), difficulty breathing, and infant hospitalization related to a breathing problem or chest infection (e.g., pneumonia)  CHW-observed axillary temperature ≥37.5 °C (confirmed with a second measurement), lower chest wall indrawing, and elevated respiratory rate (≥60 breaths per minute for infants up to 59 days of age or ≥50 breaths per minute for infants 60 days of age or older) | Caregiver-reported cough, rhinorrhea (i.e., runny nose), nasal congestion (i.e., stuffy nose), difficulty breathing, feels hot to the touch, and infant hospitalization |
| Criteria for notifying the nasal swab dispatcher | Identification of any one clinical feature indicative of a possible ARI | Any sign or symptom of ARI reported by the caregiver |
| Criteria for obtaining a nasal swab | Meets clinical case definition of URTI and/or LRTI; and  No nasal swab collected within the past 7 days; and  At least one study visit at which URTI and LRTI were absent, or if not, the ARI clinical case definition has worsened since the last nasal swab was collected | Meets clinical case definition of URTI and/or LRTI during in-person verification; and  No nasal swab collected within the past 7 days; and  At least one study visit at which UTRI and LRTI were absent, or if not, the ARI clinical case definition has worsened since the last nasal swab was collected |
| ARI, acute respiratory infection; CHW, community health worker, URTI, upper respiratory tract infection; LRTI, lower respiratory tract infection | | |

**Table S3. Comparison of mothers enrolled in the MDIG trial versus mothers whose infant is enrolled in the MDARI study**

|  | **MDIG** | **MDARI** |
| --- | --- | --- |
| **Characteristics** | **n = 1300** | **n = 1174** |
| Maternal age (y), median (minimum, maximum) | 22 (18, 18) | 23 (18, 18) |
| Gestational age (week) at enrollment, median (minimum, maximum) | 20.3 (17.0, 24.0) | 20.3 (17.0, 24.0) |
| Marital status, n (%) ^a^ |  |  |
| Married | 1283 (99.8) | 1170 (99.8) |
| Not Married | 2 (0.2) | 2 (0.2) |
| Level of education, n (%) |  |  |
| No schooling | 58 (4) | 47 (4) |
| Primary incomplete | 277 (21) | 250 (21) |
| Primary complete | 179 (14) | 165 (14) |
| Secondary incomplete | 500 (38) | 449 (38) |
| Secondary complete or higher | 286 (22) | 263 (22) |
| Primary occupation, n (%) ^a^ |  |  |
| Homemaker | 1204 (94) | 1096 (94) |
| Other | 81 (6) | 76 (6) |
| Asset index quintiles, n (%) ^b,d^ |  |  |
| 1 (lowest) | 262 (20) | 237 (20) |
| 2 | 251 (20) | 225 (19) |
| 3 | 257 (20) | 241 (21) |
| 4 | 257 (20) | 231 (20) |
| 5 (highest) | 255 (20) | 235 (20) |
| Month of enrolment, n (%) |  |  |
| March-May | 466 (36) | 418 (36) |
| June-August | 413 (32) | 374 (32) |
| September-November | 225 (17) | 206 (18) |
| December-February | 196 (15) | 176 (15) |
| Serum 25(OH)D concentration (nmol/L) at enrolment, mean ± SD ^c^ | 27.5 ± 14.0 | 27.4 ± 14.1 |

^a^ n = 1285 for MDIG trial, n = 1172 for MDARI trial

^b^ n = 1282 for MDIG trial, n = 1169 for MDARI trial

^c^ n = 1291 for MDIG trial, n = 1169 for MDARI trial

^d^ An asset index was constructed using data collected from a baseline survey on household characteristics. Ownership (yes/no) of the following 19 items was self-reported by trial participants: private toilet, electricity, radio, TV, mobile phone, non-mobile phone, fridge, almirah/wardrobe, table, chair, electric fan, DVD/CD player, autobike, rickshaw/van, bicycle, motorcycle/motor scooter/tempo/CNG, livestock/herds/farm animals/poultry, homestead, and land.

**Table S4. Supplementation duration and adherence among mothers, by supplementation group**

|  | **Prenatal; Postpartum Vitamin D Dose (IU/Week)** | | | | | **p ^a^** |
| --- | --- | --- | --- | --- | --- | --- |
|  | **0; 0** | **4200; 0** | **16800; 0** | **28000; 0** | **28000; 28000** |  |
| **Participants, N** | **234** | **239** | **233** | **233** | **235** |  |
| **Prenatal adherence** |  |  |  |  |  |  |
| Completed weekly monitoring visits, median (IQR) | 17 (16, 19) | 18 (16, 20) | 18 (16, 19) | 18 (16, 19) | 18 (16, 20) | 0.415 |
| Total supplement doses administered, median (IQR) | 19 (17, 20) | 19 (17, 21) | 19 (17, 21) | 19 (17, 21) | 19 (17, 21) | 0.241 |
| Total vitamin D administered (×1,000 IU), median (IQR) | 0 (0,0) | 80 (71, 88) | 319 (286, 353) | 532 (476, 588) | 532 (476, 588) | - |
| Adherence (%) ^b^, median (IQR) | 100 (100, 100) | 100 (100, 100) | 100 (100, 100) | 100 (100, 100) | 100 (100, 100) | 0.251 |
| Participants (%) who received |  |  |  |  |  |  |
| 100% of scheduled doses | 205 (88) | 211 (88) | 207 (89) | 197 (85) | 214 (91) | 0.293 |
| ≥90% of scheduled doses | 226 (97) | 229 (96) | 224 (96) | 215 (92) | 229 (97) | 0.065 |
| ≥80% of scheduled doses | 228 (97) | 232 (97) | 228 (98) | 219 (94) | 231 (98) | 0.058 |
| Percentage of tablets consumed under direct observation, median (IQR) | 91 (88, 95) | 90 (86, 95) | 93 (87, 95) | 91 (86, 95) | 91 (88, 95) | 0.811 |
| **Postpartum adherence** |  |  |  |  |  |  |
| Completed weekly monitoring visits, median (IQR) | 24 (23, 25) | 24 (22, 25) | 24 (22, 25) | 24 (23, 25) | 24 (23, 25) | 0.769 |
| Total supplement doses administered, median (IQR) | 26 (26, 26) | 26 (25, 26) | 26 (26, 26) | 26 (25, 26) | 26 (25, 26) | 0.137 |
| Total vitamin D administered (×1,000 IU), median (IQR) | 0 (0,0) | 0 (0, 0) | 0 (0, 0) | 0 (0, 0) | 728 (700, 728) | - |
| Adherence (%), median (IQR) | 100 (100, 100) | 100 (96, 100) | 100 (100, 100) | 100 (96, 100) | 100 (96, 100) | 0.277 |
| Participants (%) who received |  |  |  |  |  |  |
| 100% of scheduled doses | 178 (76) | 171 (72) | 179 (77) | 161 (69) | 174 (74) | 0.294 |
| ≥90% of scheduled doses | 208 (89) | 195 (82) | 203 (87) | 193 (83) | 204 (87) | 0.124 |
| ≥80% of scheduled doses | 214 (92) | 207 (87) | 212 (91) | 208 (89) | 212 (90) | 0.437 |
| Percentage of tablets consumed under direct observation, median (IQR) ^c^ | 96 (88, 96) | 92 (88, 96) | 93 (88, 96) | 96 (88, 100) | 92 (88, 96) | 0.199 |

^a^ p-values from ANOVA or Kruskal-Wallis tests for continuous variables, and Chi-square or Fischer’s tests for categorical variables. Where the overall P-value was significant, P-values for pairwise tests were adjusted for multiple comparisons using Holm test; however, they were not adjusted for the multiplicity of secondary outcomes.

^b^ Proportion of scheduled doses that were received.

^c^ N_0; 0_ = 234, N_4200; 0_ = 239, N_16800; 0_ = 232, N_28000; 0_ = 230, N_28000; 28000_ = 235

**Table S5. Maternal and infant 25-hydroxyvitamin D concentrations among mother-infant pairs enrolled in the MDARI study, overall and by supplementation group**

|  | **Prenatal; Postpartum Dose (IU/Week)** | | | | | |
| --- | --- | --- | --- | --- | --- | --- |
|  | **Overall ^a^** | **0; 0** | **4200; 0** | **16800; 0** | **28000; 0** | **28000; 28000** |
| Maternal Baseline |  |  |  |  |  |  |
| N | 1169 | 233 | 238 | 232 | 232 | 234 |
| 25(OH)D (nmol/L), mean ± SD | 27.4 ± 14.1 | 27.2 ± 13.9 | 27.6 ± 14.4 | 28.4 ± 14.0 | 27.4 ± 15.1 | 26.4 ± 13.3 |
| <30 nmol/L, n (%) | 746 (64) | 145 (62) | 147 (62) | 143 (62) | 149 (64) | 162 (69) |
| <50 nmol/L, n (%) | 1088 (93) | 220 (94) | 227 (95) | 211 (91) | 215 (93) | 215 (92) |
| Maternal Delivery |  |  |  |  |  |  |
| N | 624 | 127 | 120 | 135 | 113 | 129 |
| 25(OH)D (nmol/L), mean ± SD | 83.7 ± 40.7 | 23.8 ± 13.9 | 69.6 ± 19.6 | 100.9 ± 23.6 | 111.1 ± 27.1 | 113.6 ± 25.7 |
| <30 nmol/L, n (%) | 98 (16) | 96 (76) | 2 (2) | 0 (0) | 0 (0) | 0 (0) |
| <50 nmol/L, n (%) | 139 (22) | 122 (96) | 14 (12) | 2 (1) | 1 (1) | 0 (0) |
| Venous Umbilical Cord |  |  |  |  |  |  |
| N | 497 | 98 | 101 | 110 | 91 | 97 |
| 25(OH)D (nmol/L), mean ± SD | 50.0 ± 26.0 | 11.9 ± 7.4 | 37.2 ± 10.4 | 59.9 ± 13.0 | 71.7 ± 16.3 | 70.0 ± 16.4 |
| <30 nmol/L, n (%) | 118 (24) | 96 (98) | 22 (22) | 0 (0) | 0 (0) | 0 (0) |
| <50 nmol/L, n (%) | 234 (47) | 98 (100) | 92 (91) | 32 (29) | 3 (3) | 9 (9) |
| Infants at 3 Months |  |  |  |  |  |  |
| N | 342 | 71 | 63 | 65 | 68 | 75 |
| 25(OH)D (nmol/L), mean ± SD | 43.6 ± 26.2 | 30.6 ± 21.9 | 32.7 ± 20.4 | 37.2 ± 21.4 | 38.7 ± 19.3 | 75.2 ± 17.2 |
| <30 nmol/L, n (%) | 135 (39) | 42 (59) | 35 (56) | 30 (46) | 28 (41) | 0 (0) |
| <50 nmol/L, n (%) | 207 (61) | 54 (76) | 50 (79) | 46 (71) | 52 (76) | 5 (7) |
| Infants at 6 Months |  |  |  |  |  |  |
| N | 389 | 82 | 80 | 76 | 80 | 71 |
| 25(OH)D (nmol/L), mean ± SD | 58.2 ± 28.9 | 51.8 ± 26.6 | 53.6 ± 25.8 | 53.9 ± 26.9 | 50.8 ± 26.0 | 83.6 ± 26.4 |
| <30 nmol/L, n (%) | 67 (17) | 18 (22) | 15 (19) | 14 (18) | 18 (23) | 2 (3) |
| <50 nmol/L, n (%) | 155 (40) | 36 (44) | 36 (45) | 35 (46) | 41 (51) | 7 (10) |

^a^ 1174 mother-infant pairs were eligible for the MDARI study

**Table S6. Delivery characteristics and pregnancy outcomes, by supplementation group**

|  | **Prenatal; Postpartum Vitamin D Dose (IU/Week)** | | | | | **p ^a^** |
| --- | --- | --- | --- | --- | --- | --- |
|  | **0; 0** | **4200; 0** | **16800; 0** | **28000; 0** | **28000; 28000** |  |
| **Participants, N** | **234** | **239** | **233** | **233** | **235** |  |
| Gestational age at birth (week), median (minimum, maximum) | 39.1 (32, 43) | 39.1 (34, 42) | 39.0 (33, 43) | 39.3 (33, 43) | 39.3 (32, 42) | 0.598 |
| Early Preterm (<32 weeks), n (%) | 0 (0) | 0 (0) | 0 (0) | 0 (0) | 0 (0) | 0.849 |
| Preterm (≥32 weeks to <37 weeks), n (%) | 21 (9) | 17 (7) | 23 (10) | 19 (8) | 17 (7) |  |
| Term (≥37 weeks to <42 weeks), n (%) | 209 (89) | 215 (90) | 207 (89) | 210 (90) | 215 (91) |  |
| Postterm (≥42 weeks), n (%) | 4 (2) | 7 (3) | 3 (1) | 4 (2) | 3 (1) |  |
| Mode of delivery, n (%) |  |  |  |  |  | 0.463 |
| Vaginal birth | 116 (50) | 102 (43) | 111 (48) | 118 (51) | 110 (47) |  |
| Caesarean section | 118 (50) | 137 (57) | 122 (52) | 115 (49) | 125 (53) |  |
| Location of delivery, n (%) |  |  |  |  |  | 0.796 |
| Hospital or clinic | 200 (85) | 208 (87) | 201 (86) | 196 (84) | 197 (84) |  |
| Home | 33 (14) | 31 (13) | 31 (13) | 37 (16) | 38 (16) |  |
| Other | 1 (0.4) | 0 (0) | 1 (0.4) | 0 (0) | 0 (0) |  |
| Infant sex, n (%) |  |  |  |  |  | 0.540 |
| Male | 109 (47) | 130 (54) | 115 (49) | 120 (52) | 118 (50) |  |
| Female | 125 (53) | 109 (46) | 118 (51) | 113 (48) | 117 (50) |  |
| Congenital anomaly, n (%) | 14 (6) | 6 (3) | 5 (2) | 7 (3) | 6 (3) | 0.120 |
| Month of birth, n (%) |  |  |  |  |  | 0.183 |
| March-May | 31 (13) | 35 (15) | 37 (16) | 24 (10) | 40 (17) |  |
| June-August | 69 (29) | 62 (26) | 61 (26) | 74 (32) | 68 (29) |  |
| September-November | 82 (35) | 71 (30) | 87 (37) | 78 (33) | 64 (27) |  |
| December-February | 52 (22) | 71 (30) | 48 (21) | 57 (24) | 63 (27) |  |
| Maternal serum 25(OH)D concentration at/near delivery (nmol/L), mean ± SD ^b^ | 23.8 ± 13.9 | 69.6 ± 19.6 | 100.9 ± 23.6 | 111.1 ± 27.1 | 113.6 ± 25.7 | <0.001 |
| Newborn anthropometry, mean ± SD ^c^ |  |  |  |  |  |  |
| Birth weight (kg) ^d^ | 2.73 ± 0.36 | 2.71 ± 0.39 | 2.72 ± 0.35 | 2.67 ± 0.34 | 2.76 ± 0.35 | 0.291 |
| Length at birth (cm) ^e^ | 47.5 ± 2.1 | 47.5 ± 1.9 | 47.4 ± 1.9 | 47.2 ± 2.1 | 47.5 ± 2.0 | 0.731 |
| Head circumference at birth (cm) ^f^ | 33.0 ± 1.3 | 33.0 ± 1.3 | 33.0 ± 1.1 | 32.9 ± 1.2 | 33.0 ± 1.1 | 0.783 |
| Gestational age/sex-standardized growth parameter, mean ± SD |  |  |  |  |  |  |
| Weight-for-age z-score at birth ^d^ | -1.11 ± 0.84 | -1.26 ± 0.89 | -1.15 ± 0.90 | -1.30 ± 0.83 | -1.13 ± 0.86 | 0.192 |
| Length-for-age z-score at birth ^e^ | -0.80 ± 1.04 | -0.94 ± 1.00 | -0.91 ± 1.05 | -1.00 ± 1.02 | -0.89 ± 0.96 | 0.534 |
| Head circumference-for-age z-score at birth ^f^ | -0.55 ± 0.95 | -0.64 ± 1.03 | -0.58 ± 0.94 | -0.71 ± 0.99 | -0.60 ± 0.91 | 0.603 |
| Term low birth weight, n (%) ^g^ | 29 (20) | 44 (28) | 34 (22) | 42 (29) | 32 (21) | 0.210 |
| Low birth weight, n (%) ^d,h^ | 40 (25) | 50 (30) | 41 (25) | 51 (32) | 39 (23) | 0.312 |
| Small for gestational age, n (%) ^d,i^ | 70 (43) | 84 (50) | 75 (46) | 82 (52) | 76 (46) | 0.502 |

^a^ p-values from ANOVA or Kruskal-Wallis tests for continuous variables, and Chi-square or Fischer’s tests for categorical variables. Where the overall P-value

was significant, P-values for pairwise tests were adjusted for multiple comparisons using Holm test; however, they were not adjusted for the multiplicity of

secondary outcomes.

^b^ N_0; 0_ = 127, N_4200; 0_ = 120, N_16800; 0_ = 135, N_28000; 0_ = 113, N_28000; 28000_ = 129

^c^ Limited to measurements obtained within 48 hours of birth.

^d^ N_0; 0_ = 162, N_4200; 0_ = 167, N_16800; 0_ = 164, N_28000; 0_ = 158, N_28000; 28000_ = 166

^e^ N_0; 0_ = 160, N_4200; 0_ = 165, N_16800; 0_ = 162, N_28000; 0_ = 157, N_28000; 28000_ = 163

^f^ N_0; 0_ = 163, N_4200; 0_ = 164, N_16800; 0_ = 165, N_28000; 0_ = 156, N_28000; 28000_ = 164

^g^ Limited to infants born ≥37 weeks to <42 weeks. N_0; 0_ = 146, N_4200; 0_ = 157, N_16800; 0_ = 152, N_28000; 0_ = 146, N_28000; 28000_ = 156

^h^ Weight < 2500 g.

^i^ Weight-for-age z-score below the 10^th^ percentile, based on the Intergrowth-21^st^ Neonatal Standards.

**Table S7. ARI data collection via active and passive surveillance systems, by supplementation group**

|  | **Prenatal; Postpartum Vitamin D Dose (IU/Week)** | | | | | **p ^a^** |
| --- | --- | --- | --- | --- | --- | --- |
|  | **0; 0** | **4200; 0** | **16800; 0** | **28000; 0** | **28000; 28000** |  |
| **Participants, N** | **234** | **239** | **233** | **233** | **235** |  |
| Infant age at start of surveillance, week, Median (IQR) | 0 (0, 2) | 0 (0, 1) | 0 (0, 2) | 0 (0, 2) | 0 (0, 3) | 0.670 |
| Number of surveillance weeks, Median (IQR) | 26 (24, 26) | 26 (25, 26) | 26 (24, 26) | 26 (23, 26) | 26 (23, 26) | 0.708 |
| **All Surveillance** |  |  |  |  |  |  |
| At least one incident episode with clinical criteria, n (%) |  |  |  |  |  |  |
| ARI | 208 (89) | 217 (91) | 209 (90) | 216 (93) | 212 (90) | 0.693 |
| URTI | 208 (89) | 217 (91) | 209 (90) | 216 (93) | 212 (90) | 0.693 |
| LRTI (non-hospitalized and hospitalized) | 41 (18) | 46 (19) | 48 (21) | 43 (18) | 29 (12) | 0.166 |
| HLRTI | 14 (6) | 12 (5) | 22 (9) | 16 (7) | 10 (4) | 0.172 |
| Swabs collected per participant, Median (IQR) | 3 (2, 4) | 3 (2, 5) | 3 (2, 5) | 3 (2, 5) | 3 (1, 4) | 0.198 |
| At least one swab collected, n (%) | 206 (88) | 218 (91) | 208 (89) | 216 (93) | 212 (90) | 0.491 |
| Total swabs collected, n | 733 | 755 | 792 | 780 | 716 | 0.293 |
| **Active Surveillance** |  |  |  |  |  |  |
| At least one incident episode with clinical criteria, n (%) |  |  |  |  |  |  |
| ARI | 187 (80) | 197 (82) | 195 (84) | 195 (84) | 185 (79) | 0.523 |
| URTI | 187 (80) | 197 (82) | 195 (84) | 195 (84) | 185 (79) | 0.523 |
| LRTI (non-hospitalized and hospitalized) | 25 (11) | 27 (11) | 26 (11) | 23 (10) | 11 (5) | 0.079 |
| HLRTI ^b^ | 8 (3) | 8 (3) | 17 (7) | 8 (3) | 5 (2) | 0.048 |
| Swabs collected per participant, Median (IQR) | 2 (1, 3) | 2 (1, 3) | 2 (1, 4) | 2 (1, 3) | 2 (1, 3) | 0.082 |
| At least one swab collected, n (%) | 183 (78) | 194 (81) | 193 (83) | 191 (82) | 183 (78) | 0.558 |
| Total swabs collected, n | 503 | 489 | 558 | 528 | 471 | 0.491 |
| Positive swabs among swabs collected, n (%) ^c^ | 108 (21) | 102 (21) | 126 (23) | 109 (21) | 110 (23) | 0.849 |
| **Passive Surveillance** |  |  |  |  |  |  |
| At least one incident episode with clinical criteria, n (%) |  |  |  |  |  |  |
| ARI | 130 (56) | 140 (59) | 130 (56) | 132 (57) | 130 (55) | 0.954 |
| URTI | 130 (56) | 140 (59) | 130 (56) | 132 (57) | 130 (55) | 0.954 |
| LRTI (non-hospitalized and hospitalized) | 24 (10) | 30 (13) | 30 (13) | 27 (12) | 21 (9) | 0.637 |
| HLRTI | 7 (3) | 5 (2) | 7 (3) | 10 (4) | 5 (2) | 0.614 |
| Swabs collected per participant, Median (IQR) | 1 (0, 2) | 1 (0, 2) | 1 (0, 2) | 1 (0, 2) | 1 (0, 2) | 0.908 |
| At least one swab collected, n (%) | 131 (56) | 141 (59) | 130 (56) | 134 (58) | 135 (57) | 0.956 |
| Total swabs collected, n | 230 | 266 | 234 | 252 | 245 | 0.967 |
| Positive swabs among swabs collected, n (%) ^c^ | 48 (21) | 61 (23) | 54 (23) | 60 (24) | 63 (26) | 0.863 |

^a^ p-values from Kruskal-Wallis tests for continuous variables, Chi-square or Fischer’s tests for categorical variables, and generalized estimating equations for correlated variables (total swabs collected and positive swabs collected). Where the overall P-value was significant, P-values for pairwise tests were adjusted for multiple comparisons using Holm test; however, they were not adjusted for the multiplicity of secondary outcomes.

^b^ Pairwise comparisons for Active Surveillance, HLRTI: 4200;0 vs 0;0: p = 0.524; 16,800;0 vs 0;0: p = 0.167; 28,000;0 vs 0;0: p = 0.994; 28,000;28,000 vs 0;0: p = 0.199 ^C^ Among total swabs collected

**Table S8. ARI reported by CHW, caregiver, and study physician, by supplementation group**

|  | **Prenatal; Postpartum Vitamin D Dose (IU/Week)** | | | | |
| --- | --- | --- | --- | --- | --- |
|  | **0;  0** | **4200;  0** | **16800;  0** | **28000;  0** | **28000;  28000** |
| **Number of weeks of surveillance** | 5446 | 5628 | 5435 | 5355 | 5355 |
| **Active surveillance** |  |  |  |  |  |
| Number of household visits | 4890 | 4907 | 4842 | 4749 | 4783 |
| Number of ARI cases reported by a CHW | 1420 | 1353 | 1416 | 1449 | 1285 |
| **Passive surveillance** |  |  |  |  |  |
| Number of ARI cases reported |  |  |  |  |  |
| By a caregiver | 83 | 98 | 83 | 83 | 78 |
| By a study physician | 181 | 198 | 174 | 194 | 184 |
| **Overlap** |  |  |  |  |  |
| Number of ARI cases reported |  |  |  |  |  |
| By a CHW, a caregiver and a study physician | 0 | 1 | 1 | 0 | 0 |
| By a CHW and a caregiver only | 44 | 64 | 39 | 44 | 36 |
| By a CHW and a study physician only | 127 | 125 | 104 | 123 | 119 |

**Table S9. Number of episodes of LRTI per infant from 0 to 6 months of age, by supplementation group**

|  | **Prenatal; Postpartum Vitamin D Dose (IU/Week)** | | | | |
| --- | --- | --- | --- | --- | --- |
|  | **0;  0** | **4200;  0** | **16800;  0** | **28000;  0** | **28000; 28000** |
|  | **(n = 234)** | **(n = 239)** | **(n = 233)** | **(n = 233)** | **(n = 235)** |
| Number of episodes of clinically diagnosed LRTI, number of infants (%) |  |  |  |  |  |
| 0 | 192 (82) | 193 (81) | 182 (78) | 188 (81) | 205 (87) |
| 1 | 28 (12) | 42 (18) | 37 (16) | 35 (15) | 23 (10) |
| 2 | 11 (5) | 3 (1) | 10 (4) | 8 (3) | 4 (2) |
| 3 | 2 (1) | 1 (0.4) | 3 (1) | 2 (1) | 3 (1) |
| 4 | 1 (0.4) | 0 (0) | 1 (0.4) | 0 (0) | 0 (0) |
| Number of episodes of microbiologically confirmed LRTI, number of infants (%) |  |  |  |  |  |
| 0 | 213 (91) | 222 (93) | 203 (87) | 209 (90) | 217 (92) |
| 1 | 20 (9) | 17 (7) | 29 (12) | 22 (9) | 17 (7) |
| 2 | 1 (0.4) | 0 (0) | 1 (0.4) | 2 (1) | 1 (0.4) |

**Table S10. Respiratory viruses identified in episodes of microbiologically confirmed ARI, by ARI type**

|  | **Microbiologically confirmed** | | |
| --- | --- | --- | --- |
|  | **ARI (n = 820)** | **URTI (n = 817)** | **LRTI (n = 115)** |
| Influenza A | 68 (8) | 68 (8) | 4 (3) |
| Influenza B | 40 (5) | 40 (5) | 0 (0) |
| Influenza A or B | 107 (13) | 107 (13) | 4 (3) |
| Parainfluenza 1 | 26 (3) | 26 (3) | 1 (1) |
| Parainfluenza 2 | 19 (2) | 19 (2) | 0 (0) |
| Parainfluenza 3 | 216 (26) | 215 (26) | 30 (26) |
| Parainfluenza 1/2/3 | 257 (31) | 256 (31) | 31 (27) |
| Adenovirus | 127 (15) | 126 (15) | 12 (10) |
| RSV | 153 (19) | 152 (19) | 49 (43) |
| hMPV | 188 (23) | 188 (23) | 32 (28) |
| All values are expressed as number (percentage) and represent the number (percentage) of microbiologically-confirmed episodes of ARI, URTI or LRTI in which the listed virus was identified.  8% of microbiologically confirmed ARI had more than one virus identified. | | | |

**Table S11. Respiratory viruses identified in episodes of clinical ARI, by ARI type**

|  | **Clinically confirmed** | | |
| --- | --- | --- | --- |
|  | **ARI (n = 3687)** | **URTI (n = 3673)** | **LRTI (n = 278)** |
| Influenza A | 68 (2) | 68 (2) | 4 (1) |
| Influenza B | 40 (1) | 40 (1) | 0 (0) |
| Influenza A or B | 107 (3) | 107 (3) | 4 (1) |
| Parainfluenza 1 | 26 (1) | 26 (1) | 1 (0.4) |
| Parainfluenza 2 | 19 (1) | 19 (1) | 0 (0) |
| Parainfluenza 3 | 216 (6) | 215 (6) | 30 (11) |
| Parainfluenza 1/2/3 | 257 (7) | 256 (7) | 31 (11) |
| Adenovirus | 127 (3) | 126 (3) | 12 (4) |
| RSV | 153 (4) | 152 (4) | 49 (18) |
| hMPV  No virus identified | 188 (5)  2913 (79) | 188 (5)  2902 (79) | 32 (12)  165 (59) |
| All values are expressed as number (percentage) and represent the number (percentage) of all episodes of ARI, URTI or LRTI (including both those with and without a virus detected) in which the listed virus was identified.  1.3% of clinically confirmed ARI had more than one virus identified. | | | |

**Table S12. Effect of mid- or high-dose prenatal vitamin D supplementation (16,800 or 28,000 IU) versus placebo (0 IU/week) or low-dose (4,200 IU/week) vitamin D supplementation on incidence of acute respiratory infections up to 6 months of age**

|  | **0; 0 or 4200; 0**  **(n = 472)** | | | **16800; 0 or 28000; 0 or 28000; 28000**  **(n = 701)** | | | **HR (95% CI)** | **p ^a^** |
| --- | --- | --- | --- | --- | --- | --- | --- | --- |
|  | **Number of incident episodes** | **Person-time at risk  (weeks)** | **Incidence rate (per 6 person-months)** | **Number of incident episodes** | **Person- time at risk (weeks)** | **Incidence rate (per 6 person-months)** |  |  |
| Microbiologically confirmed |  |  |  |  |  |  |  |  |
| ARI | 309 | 7464 | 1.08 | 511 | 10834 | 1.23 | 1.13 (0.98, 1.30) | 0.083 |
| URTI | 309 | 7450 | 1.08 | 508 | 10823 | 1.22 | 1.12 (0.98, 1.29) | 0.101 |
| LRTI ^b^ | 39 | 9409 | 0.11 | 76 | 13788 | 0.14 | 1.32 (0.90, 1.96) | 0.160 |
| HLRTI | 18 | 9491 | 0.05 | 37 | 13902 | 0.07 | 1.40 (0.79, 2.46) | 0.246 |
| ARI with RSV | 66 | 7464 | 0.23 | 107 | 10834 | 0.26 | 1.11 (0.81, 1.53) | 0.505 |
| ARI with Influenza A/B | 42 | 7464 | 0.15 | 68 | 10834 | 0.16 | 1.10 (0.74, 1.64) | 0.627 |
| Clinical criteria |  |  |  |  |  |  |  |  |
| ARI | 1454 | 7464 | 5.06 | 2233 | 10832 | 5.36 | 1.05 (0.97, 1.15) | 0.243 |
| URTI | 1449 | 7449 | 5.06 | 2224 | 10822 | 5.34 | 1.05 (0.96, 1.15) | 0.258 |
| LRTI ^b^ | 111 | 9408 | 0.31 | 167 | 13785 | 0.31 | 1.03 (0.77, 1.36) | 0.860 |
| HLRTI | 29 | 9490 | 0.08 | 57 | 13899 | 0.11 | 1.34 (0.85, 2.12) | 0.213 |

^a^ p-values from a cox proportional hazards model. Jackknife estimation of standard errors was used to account for repeated events within the same infant

^b^ Non-hospitalized and hospitalized cases of lower respiratory tract infection

**Table S13. Period prevalence of microbiologically confirmed ARI and clinical ARI among infants from 0 to 6 months of age, by supplementation group**

|  | **Number of prevalent weeks** | | **Period prevalence ratio**  **(95% CI)** | **p ^a^** |
| --- | --- | --- | --- | --- |
|  | **0; 0** | **28000; 28000** |  |  |
|  | **(n = 234)** | **(n = 235)** |  |  |
| Total person-time observed, weeks | 5,446 | 5,335 |  |  |
| Microbiologically confirmed |  |  |  |  |
| ARI | 253 | 274 | 1.10 (0.86, 1.42) | 0.434 |
| URTI | 252 | 274 | 1.11 (0.86, 1.42) | 0.418 |
| LRTI ^b^ | 28 | 22 | 0.82 (0.41, 1.61) | 0.557 |
| HLRTI | 13 | 10 | 0.79 (0.27, 2.28) | 0.661 |
| ARI with RSV | 48 | 64 | 1.35 (0.78, 2.34) | 0.287 |
| ARI with Influenza A/B | 36 | 42 | 1.19 (0.60, 2.34) | 0.623 |
| Clinical criteria |  |  |  |  |
| ARI | 1126 | 1060 | 0.97 (0.85, 1.10) | 0.653 |
| URTI | 1124 | 1059 | 0.97 (0.85, 1.11) | 0.661 |
| LRTI ^b^ | 64 | 44 | 0.70 (0.41, 1.17) | 0.169 |
| HLRTI | 18 | 13 | 0.72 (0.31, 1.72) | 0.465 |

^a^ From a generalized estimating equation for log-binomial model with robust standard errors to account for repeated observations within the same infant

^b^ Non-hospitalized and hospitalized cases of lower respiratory tract infection

**Table S14. Effect of 28,000 IU vitamin D per week versus placebo in the postpartum period on incidence of infant acute respiratory infections among infants born to women administered 28,000 IU/week vitamin D in the prenatal period**

|  | **28000; 0 (n = 233)** | | | **28000; 28000 (n = 235)** | | | **HR (95% CI)** | **p ^a^** |
| --- | --- | --- | --- | --- | --- | --- | --- | --- |
|  | **Number of incident episodes** | **Person-time at risk  (weeks)** | **Incidence rate (per 6 person-months)** | **Number of incident episodes** | **Person- time at risk (weeks)** | **Incidence rate (per 6 person-months)** |  |  |
| Microbiologically confirmed |  |  |  |  |  |  |  |  |
| ARI | 166 | 3526 | 1.22 | 171 | 3679 | 1.21 | 0.98 (0.78, 1.23) | 0.870 |
| URTI | 163 | 3522 | 1.20 | 171 | 3676 | 1.21 | 1.00 (0.80, 1.25) | 0.995 |
| LRTI ^b^ | 26 | 4551 | 0.15 | 19 | 4604 | 0.11 | 0.73 (0.39, 1.36) | 0.314 |
| HLRTI | 11 | 4590 | 0.06 | 8 | 4634 | 0.04 | 0.73 (0.28, 1.88) | 0.509 |
| ARI with RSV | 36 | 3526 | 0.27 | 36 | 3679 | 0.25 | 0.95 (0.58, 1.56) | 0.848 |
| ARI with Influenza A/B | 27 | 3526 | 0.20 | 26 | 3679 | 0.18 | 0.91 (0.51, 1.62) | 0.747 |
| Clinical criteria |  |  |  |  |  |  |  |  |
| ARI | 761 | 3525 | 5.61 | 704 | 3679 | 4.98 | 0.88 (0.77, 1.01) | 0.065 |
| URTI | 758 | 3521 | 5.60 | 703 | 3677 | 4.97 | 0.88 (0.77, 1.01) | 0.069 |
| LRTI ^b^ | 57 | 4550 | 0.33 | 40 | 4604 | 0.23 | 0.69 (0.42, 1.14) | 0.145 |
| HLRTI | 19 | 4589 | 0.11 | 11 | 4634 | 0.06 | 0.58 (0.27, 1.24) | 0.160 |

^a^ p-values from a cox proportional hazards model. Jackknife estimation of standard errors was used to account for repeated events within the same infant

^b^ Non-hospitalized and hospitalized cases of lower respiratory tract infection

**Table S15. Cumulative incidence of at least one episode of ARI, by supplementation group**

|  | **Prenatal; Postpartum Vitamin D Dose (IU/Week)** | | | | | **p ^a^** |
| --- | --- | --- | --- | --- | --- | --- |
|  | **0; 0** | **4200; 0** | **16800; 0** | **28000; 0** | **28000; 28000** |  |
| **Participants, N** | **233** | **239** | **233** | **233** | **235** |  |
| **Microbiologically confirmed, n (%)** |  |  |  |  |  |  |
| ARI | 118 (50) | 121 (51) | 133 (57) | 120 (52) | 124 (53) | 0.592 |
| URTI | 118 (50) | 121 (51) | 133 (57) | 119 (51) | 124 (53) | 0.576 |
| LRTI ^b^ | 21 (9) | 17 (7) | 30 (13) | 24 (10) | 18 (8) | 0.207 |
| HLRTI | 10 (4) | 8 (3) | 18 (8) | 11 (5) | 8 (3) | 0.151 |
| ARI with RSV | 33 (14) | 28 (12) | 31 (13) | 31 (13) | 32 (14) | 0.955 |
| ARI with Influenza A/B | 19 (8) | 21 (9) | 15 (6) | 24 (10) | 24 (10) | 0.558 |
| **Clinical criteria, n (%)** |  |  |  |  |  |  |
| ARI | 208 (89) | 217 (91) | 209 (90) | 216 (93) | 212 (90) | 0.693 |
| URTI | 208 (89) | 217 (81) | 209 (90) | 216 (93) | 212 (90) | 0.693 |
| LRTI ^b^ | 42 (18) | 46 (19) | 51 (22) | 45 (19) | 30 (13) | 0.128 |
| HLRTI | 16 (7) | 12 (5) | 25 (11) | 18 (8) | 11 (5) | 0.073 |

^a^ p-values from Chi-square tests

^b^ Non-hospitalized and hospitalized cases of lower respiratory tract infection

**Table S16. Caregiver-reported and study worker-observed clinical signs and identified hospitalization among incident ARI cases, by supplementation group**

|  | **Incident ARI cases meeting clinical criteria only (n = 3693)** | | | | | | | **Microbiologically confirmed incident ARI cases (n = 817)** | | | | | | |
| --- | --- | --- | --- | --- | --- | --- | --- | --- | --- | --- | --- | --- | --- | --- |
|  | **0;  0** | **4200;  0** | **16800;  0** | **28000;  0** | **28000;  28000** | **28000; 28000 vs. 0; 0** | | **0;  0** | **4200;  0** | **16800;  0** | **28000;  0** | **28000;  28000** | **28000; 28000 vs. 0; 0** | |
|  | **(n = 724)** | **(n = 730)** | **(n = 768)** | **(n = 761)** | **(n = 704)** | **OR (95% CI)** | **p ^a^** | **(n = 154)** | **(n = 155)** | **(n = 174)** | **(n = 166)** | **(n = 171)** | **OR (95% CI)** | **p ^a^** |
| Caregiver-reported signs, n (%) |  |  |  |  |  |  |  |  |  |  |  |  |  |  |
| Runny nose | 614 (85) | 639 (88) | 667 (87) | 661 (87) | 585 (83) | 0.90 (0.65, 1.24) | 0.516 | 138 (90) | 143 (92) | 155 (89) | 146 (88) | 148 (87) | 0.80 (0.40, 1.61) | 0.534 |
| Nasal congestion | 634 (88) | 624 (85) | 679 (88) | 666 (88) | 603 (86) | 0.80 (0.56, 1.13) | 0.204 | 134 (87) | 125 (81) | 156 (90) | 144 (87) | 144 (84) | 0.71 (0.37, 1.38) | 0.313 |
| Cough | 632 (87) | 644 (88) | 674 (88) | 672 (88) | 612 (87) | 0.97 (0.69, 1.36) | 0.861 | 143 (93) | 145 (94) | 164 (94) | 157 (95) | 162 (95) | 1.24 (0.56, 2.77) | 0.594 |
| Difficulty breathing | 27 (4) | 13 (2) | 41 (5) | 11 (1) | 19 (3) | 0.72 (0.39, 1.31) | 0.278 | 14 (9) | 5 (3) | 19 (11) | 7 (4) | 11 (6) | 0.68 (0.29, 1.57) | 0.367 |
| Study worker-observed signs and identified hospitalization, n (%) |  |  |  |  |  |  |  |  |  |  |  |  |  |  |
| Chest in-drawing | 41 (6) | 36 (5) | 46 (6) | 38 (5) | 31 (4) | 0.76 (0.43, 1.33) | 0.332 | 17 (11) | 12 (8) | 23 (13) | 18 (11) | 16 (9) | 0.83 (0.40, 1.72) | 0.615 |
| Elevated respiratory rate | 20 (3) | 26 (4) | 33 (4) | 25 (3) | 20 (3) | 1.03 (0.51, 2.07) | 0.942 | 9 (6) | 10 (6) | 16 (9) | 13 (8) | 8 (5) | 0.75 (0.27, 2.09) | 0.578 |
| Fever | 21 (3) | 19 (3) | 18 (2) | 17 (2) | 22 (3) | 1.09 (0.58, 2.06) | 0.791 | 4 (3) | 7 (5) | 6 (3) | 6 (4) | 11 (6) | 2.60 (0.82, 8.28) | 0.106 |
| Hospitalization | 14 (2) | 12 (2) | 25 (3) | 17 (2) | 10 (1) | 0.74 (0.34, 1.61) | 0.443 | 9 (6) | 8 (5) | 17 (10) | 11 (7) | 8 (5) | 0.79 (0.31, 2.01) | 0.620 |

^a^ p-values comparing counts in the 28000; 28000 supplementation group vs 0; 0 supplementation group using GEE for logistic regression with Binomial distribution to adjust for clustering at the infant level

**Table S17. Unadjusted and adjusted analyses of associations between participant or household characteristics and incidence of microbiologically confirmed acute respiratory infections in infants from 0 to 6 months of age**

|  | **Unadjusted** | | | **Adjusted ^a^ (n = 469)** | |
| --- | --- | --- | --- | --- | --- |
|  | **n** | **HR (95% CI)** | **p ^b^** | **HR (95% CI)** | **p ^b^** |
| Treatment group |  |  |  |  |  |
| 0; 0 | 234 | ref |  | Ref |  |
| 28000; 28000 | 235 | 1.12 (0.90, 1.40) | 0.315 | 1.13 (0.90, 1.43) | 0.271 |
| Household smoking |  |  |  |  |  |
| Never | 769 | ref |  | Ref |  |
| Ever | 400 | 1.17 (1.01, 1.35) | 0.032 | 1.04 (0.81, 1.34) | 0.732 |
| Location of cooking facilities;  type of fuel |  |  |  |  |  |
| In the home; solid fuel | 35 | ref |  | Ref |  |
| In the; non-solid fuel | 1121 | 0.78 (0.53, 1.15) | 0.212 | 0.74 (0.39, 1.41) | 0.360 |
| Outside the home; any fuel | 14 | 1.22 (0.61, 2.47) | 0.570 | 0.56 (0.10, 3.16) | 0.508 |
| Source of water |  |  |  |  |  |
| Other | 928 | ref |  | Ref |  |
| Piped | 239 | 1.08 (0.92, 1.26) | 0.350 | 0.972 (0.74, 1.27) | 0.832 |
| Mother's level of education |  |  |  |  |  |
| No schooling | 47 | ref |  | Ref |  |
| Primary incomplete | 250 | 0.75 (0.54, 1.04) | 0.082 | 0.88 (0.38, 2.05) | 0.774 |
| Primary complete | 165 | 0.69 (0.49, 0.98) | 0.036 | 0.68 (0.28, 1.62) | 0.384 |
| Secondary incomplete | 449 | 0.71 (0.52, 0.97) | 0.031 | 0.91 (0.39, 2.10) | 0.821 |
| Secondary complete or higher | 263 | 0.57 (0.41, 0.78) | 0.001 | 0.72 (0.30, 1.71) | 0.456 |
| Number of children by same mother |  |  |  |  |  |
| 1 | 549 | ref |  | Ref |  |
| More than 1 | 625 | 1.28 (1.11, 1.47) | 0.001 | 1.10 (0.87, 1.40) | 0.832 |
| Asset index quintile |  |  |  |  |  |
| 1 (lowest) | 237 | ref |  | Ref |  |
| 2 | 225 | 1.00 (0.80, 1.24) | 0.987 | 1.00 (0.67, 1.49) | 0.989 |
| 3 | 241 | 0.92 (0.74, 1.14) | 0.450 | 0.95 (0.66, 1.35) | 0.759 |
| 4 | 231 | 0.92 (0.74, 1.14) | 0.466 | 1.11 (0.76, 1.61) | 0.583 |
| 5 (highest) | 235 | 0.84 (0.68, 1.04) | 0.109 | 0.99 (0.68, 1.45) | 0.968 |

^a^ All hazard ratios are from a model in which the variables are mutually adjusted for all other covariates listed in the table.

^b^ p-values correspond to the HR for treatment group from Cox proportional hazards model with the Anderson-Gill extension, using jackknife estimation of standard errors to account for repeated events within the same infant.

**Table S18. Effect of maternal 28,000 IU/week vitamin D supplementation in prenatal and postpartum periods versus placebo, on the incidence of microbiologically confirmed acute respiratory infections in infants from 0 to 6 months of age, by sex, season, month and breastfeeding status**

| **Stratification variable** | **Strata** | **Number of subjects** | **Number of events** | **HR (95% CI)** | **p ^a^** | **p_Int_ ^b^** |
| --- | --- | --- | --- | --- | --- | --- |
| **28000; 28000 versus 0; 0** | | |  |  |  |  |
| - | Overall | 469 | 325 | 1.12 (0.90, 1.40) | 0.315 | - |
| Sex | Boy | 227 | 152 | 1.01 (0.73, 1.40) | 0.956 | 0.351 |
|  | Girl | 242 | 173 | 1.24 (0.92, 1.69) | 0.163 |  |
| Season | Low season | 279 | 70 | 1.27 (0.80, 2.02) | 0.310 | 0.404 |
|  | High season | 465 | 255 | 1.07 (0.84, 1.38) | 0.575 |  |
| Age | 0 to <2 months | 387 | 37 | 1.89 (0.95, 3.73) | 0.068 | - |
|  | 2 to 6 months | 465 | 288 | 1.05 (0.83, 1.33) | 0.682 |  |
| Breastfeeding status | Non-predominant | 100 | 64 | 1.25 (0.72, 2.17) | 0.431 | 0.684 |
|  | Exclusive/Predominant | 369 | 261 | 1.09 (0.85, 1.39) | 0.503 |  |

^a^ p-values correspond to the HR for treatment group from stratified Cox proportional hazards model with the Anderson-Gill extension, using jackknife estimation of standard errors to account for repeated events within the same infant.

^b^ p-values correspond to the HR for the product term between the treatment group and the stratification variable from Cox proportional hazards model with the Anderson-Gill extension, using jackknife estimation of standard errors to account for repeated events within the same infant.

**Table S19. Sensitivity analyses of the effect of high-dose maternal prenatal and postpartum vitamin D supplementation (28,000 IU/week) versus placebo on incidence of microbiologically confirmed acute respiratory infections in infants from 0 to 6 months of age**

|  | **0; 0** | | | | |  | **28000; 28000** | | | | **HR (95% CI)** | **p ^a^** |
| --- | --- | --- | --- | --- | --- | --- | --- | --- | --- | --- | --- | --- |
|  | **Number of infants** | **Number of incident episodes** | **Person-time at risk (weeks)** | **Incidence rate (per 6 person-months)** | **Time to first episode (weeks), median (IQR)** | **Number of infants** | **Number of incident episodes** | **Person- time at risk (weeks)** | **Incidence rate (per 6 person-months)** | **Time to first episode in (weeks),  median (IQR)** |  |  |
| Primary analysis | 234 | 154 | 3728 | 1.07 | 17 (13, 22) | 235 | 171 | 3679 | 1.21 | 17 (10, 21) | 1.12 (0.90, 1.40) | 0.315 |
| Variations to the at-risk definition | | | | | | | | | | | | |
| Allowing infants to be at-risk during week 1 ^b^ | 235 | 154 | 3891 | 1.03 | 17 (13, 22) | 235 | 171 | 3828 | 1.16 | 17 (10, 21) | 1.12 (0.90, 1.40) | 0.315 |
| Allowing infants to be at-risk in weeks with incomplete household visits ^c^ | 236 | 154 | 3800 | 1.05 | 17 (13, 22) | 235 | 171 | 3762 | 1.18 | 17 (10, 21) | 1.12 (0.90, 1.40) | 0.316 |
| Not allowing infants to be at risk during the second week of life ^d^ | 234 | 153 | 3573 | 1.11 | 17 (13, 22) | 235 | 170 | 3536 | 1.25 | 17 (10, 21) | 1.12 (0.90, 1.40) | 0.317 |
| Restricted to infants enrolled in MDARI by 4 weeks of age | 180 | 120 | 3159 | 0.99 | 17 (13, 22) | 178 | 135 | 3138 | 1.12 | 16 (9, 21) | 1.12 (0.87, 1.44) | 0.385 |
| Removing requirement of an observed illness-free week for an infant to be considered at-risk | 234 | 157 | 4753 | 0.86 | 17.5 (13, 22) | 235 | 174 | 4646 | 0.97 | 17.5 (10, 21) | 1.14 (0.92, 1.40) | 0.227 |
| Allowing participants to be at-risk during all weeks while enrolled in MDARI | 236 | 157 | 5498 | 0.74 | 17.5 (13, 22) | 235 | 174 | 5355 | 0.84 | 17.5 (10, 21) | 1.13 (0.91, 1.40) | 0.257 |
| A household visit is considered complete only if the infant can be examined ^e^ | 234 | 154 | 3720 | 1.08 | 17 (13, 22) | 235 | 171 | 3677 | 1.21 | 17 (10, 21) | 1.12 (0.90, 1.40) | 0.318 |
| Case definition |  |  |  |  |  |  |  |  |  |  |  |  |
| Including only infants whose mothers met the prenatal per-protocol criteria | 220 | 148 | 3533 | 1.09 | 17 (13, 22) | 209 | 161 | 3305 | 1.27 | 17 (10, 21) | 1.15 (0.91, 1.44) | 0.237 |
| Including only infants whose mothers met the postpartum per-protocol criteria | 182 | 121 | 2970 | 1.06 | 17.5 (13, 22) | 176 | 133 | 2981 | 1.16 | 17 (9, 21) | 1.10 (0.85, 1.41) | 0.463 |
| Including only infants whose mothers met the complete per-protocol criteria | 177 | 119 | 2884 | 1.07 | 17 (13, 22) | 165 | 131 | 2785 | 1.22 | 16 (9, 21) | 1.14 (0.89, 1.47) | 0.309 |
| Defining prevalence of clinical ARI based on reported symptoms ^f^ | 234 | 154 | 3730 | 1.07 | 17 (13, 22) | 235 | 170 | 3682 | 1.20 | 17 (10, 21) | 1.11 (0.89, 1.39) | 0.341 |
| Surveillance |  |  |  |  |  |  |  |  |  |  |  |  |
| Excluding partial weeks ^g^ | 234 | 151 | 3587 | 1.09 | 17 (13, 22) | 235 | 168 | 3529 | 1.24 | 17 (10, 21) | 1.13 (0.90, 1.41) | 0.289 |
| Accounting for duration of week in analysis ^g^ | 234 | 154 | 3686 | 1.09 | 17 (13, 22) | 235 | 171 | 3635 | 1.22 | 17 (10, 21) | 1.11 (0.89, 1.39) | 0.338 |
| Defining the duration of person-time at risk during a week with an incident ARI event as half of a week | 234 | 154 | 3651 | 1.10 | 17 (13, 22) | 235 | 171 | 3594 | 1.24 | 17 (10, 21) | 1.12 (0.90, 1.40) | 0.315 |
| Including ARI cases detected only through active surveillance | 234 | 108 | 3800 | 0.74 | 19 (15, 24) | 235 | 108 | 3770 | 0.74 | 19 (13, 23) | 1.00 (0.76, 1.33) | 0.992 |
| Swab |  |  |  |  |  |  |  |  |  |  |  |  |
| Excluding swabs that were not collected in the same week as the time of incidence ^h^ | 234 | 145 | 3728 | 1.01 | 18 (13, 22) | 235 | 164 | 3679 | 1.16 | 18 (11, 21) | 1.14 (0.91, 1.43) | 0.248 |
| Including only swabs that were taken on the same day as when the study team were notified of the ARI | 234 | 146 | 3728 | 1.02 | 17 (13, 21.5) | 235 | 168 | 3679 | 1.19 | 17 (10, 21) | 1.16 (0.92, 1.45) | 0.199 |

^a^ p-values correspond to the HR for treatment group from Cox proportional hazards model with the Anderson-Gill extension, using jackknife estimation of standard errors to account for repeated events within the same infant.

^b^ In the primary analysis, infants are not considered at risk during the first week of life ARI symptoms during this week are common in infants but are rarely indicative of actual ARI

^c^ Weeks in which a household visit was not completed and the study team were not alerted of an ARI event (either by a study physician or by a caregiver), then the infant was considered to not be under observation, and therefore not at risk of ARI

^d^ Since the first week of life was not considered to be at risk of ARI, the second week was set to always be at risk (as long as the infant was under observation during that week)

^e^ In the primary analysis, a household visit was considered complete if the infant was available for examination or if a caregiver was available to answer questions about the presence of ARI symptoms in the infant

^f^ There were some cases in which there was a discrepancy between prevalence of ARI as indicated by a CHW, and the prevalence of symptoms reported. In the primary analysis, the prevalence for ARI as marked by the CHW was considered the gold standard.

^g^ Based on the timing of the weekly visits, there were some instances in which visit occurred less than 7 days apart. Therefore, the duration of certain “weeks” were less than 7 days.

^h^ A positive swab was deemed to apply for the duration of an ARI episode. For example, if a swab was taken and the result was negative but a later swab taken in the same ARI episode tested positive, then the incident case of ARI was considered microbiologically positive.

**Table S20. Effect of high-dose maternal prenatal vitamin D supplementation (28,000 IU/week) with or without postpartum vitamin D (0 IU/week or 28,000 IU/week) versus placebo on incidence of microbiologically confirmed acute respiratory infections in infants from 0 to 6 months of age**

|  | **0; 0**  **(n = 234)** | | | | **28000;0 and 28000; 28000**  **(n = 468)** | | | | **HR (95% CI)** | **p ^a^** |
| --- | --- | --- | --- | --- | --- | --- | --- | --- | --- | --- |
| **Microbiologically confirmed** | **Number of incident episodes** | **Person-time at risk (weeks)** | **Incidence rate (per 6 person-months)** | **Time to first episode (weeks), median (IQR)** | **Number of incident episodes** | **Person- time at risk (weeks)** | **Incidence rate (per 6 person-months)** | **Time to first episode in (weeks),  median (IQR)** |  |  |
| ARI | 154 | 3728 | 1.07 | 17 (13, 22) | 337 | 7205 | 1.22 | 17 (11, 21) | 1.13 (0.93, 1.37) | 0.215 |
| URTI | 154 | 3720 | 1.08 | 17 (13, 22) | 334 | 7198 | 1.21 | 17 (11, 21) | 1.12 (0.92, 1.36) | 0.250 |
| LRTI ^b^ | 22 | 4693 | 0.12 | 15 (13, 23) | 45 | 9155 | 0.13 | 14 (9, 18) | 1.04 (0.61, 1.77) | 0.885 |
| HLRTI | 10 | 4736 | 0.05 | 22 (15, 25) | 19 | 9224 | 0.05 | 15 (9, 19) | 0.97 (0.45, 2.13) | 0.947 |

^a^ p-values from a cox proportional hazards model. Jackknife estimation of standard errors was used to account for repeated events within the same infant

^b^ Non-hospitalized and hospitalized cases of lower respiratory tract infection

**Table S21. Effect of maternal prenatal vitamin D supplementation of either 16,800 or 28,000 IU/week (with or without 28,000 IU/week in the postpartum period), versus placebo, on incidence of microbiologically confirmed acute respiratory infections in infants from 0 to 6 months of age**

|  | **0; 0**  **(n = 234)** | | | | **16800;0, 28000;0 and 28000; 28000**  **(n = 701)** | | | | **HR (95% CI)** | **p ^a^** |
| --- | --- | --- | --- | --- | --- | --- | --- | --- | --- | --- |
| **Microbiologically confirmed** | **Number of incident episodes** | **Person-time at risk (weeks)** | **Incidence rate (per 6 person-months)** | **Time to first episode (weeks), median (IQR)** | **Number of incident episodes** | **Person- time at risk (weeks)** | **Incidence rate (per 6 person-months)** | **Time to first episode in (weeks),  median (IQR)** |  |  |
| ARI | 154 | 3728 | 1.07 | 17 (13, 22) | 511 | 10834 | 1.23 | 17 (10, 21) | 1.14 (0.95, 1.37) | 0.158 |
| URTI | 154 | 3720 | 1.08 | 17 (13, 22) | 508 | 10823 | 1.22 | 17 (10, 21) | 1.13 (0.94, 1.36) | 0.178 |
| LRTI ^b^ | 22 | 4693 | 0.12 | 15 (13, 23) | 76 | 13788 | 0.14 | 14.5 (9.5, 21.5) | 1.17 (0.72, 1.91) | 0.532 |
| HLRTI | 10 | 4736 | 0.05 | 22 (15, 25) | 37 | 13902 | 0.07 | 16 (12, 22) | 1.26 (0.62, 2.58) | 0.521 |

^a^ p-values from a cox proportional hazards model. Jackknife estimation of standard errors was used to account for repeated events within the same infant

^b^ Non-hospitalized and hospitalized cases of lower respiratory tract infection

**Table S22. Effect of any dose of prenatal or postpartum maternal vitamin D supplementation versus placebo on incidence of microbiologically confirmed acute respiratory infections in infants from 0 to 6 months of age**

|  | **0; 0**  **(n = 234)** | | | | **4200;0, 16400;0, 28000;0 and 28000; 28000**  **(n = 940)** | | | | **HR (95% CI)** | **p ^a^** |
| --- | --- | --- | --- | --- | --- | --- | --- | --- | --- | --- |
| **Microbiologically confirmed** | **Number of incident episodes** | **Person-time at risk (weeks)** | **Incidence rate (per 6 person-months)** | **Time to first episode (weeks), median (IQR)** | **Number of incident episodes** | **Person- time at risk (weeks)** | **Incidence rate (per 6 person-months)** | **Time to first episode in (weeks),  median (IQR)** |  |  |
| ARI | 154 | 3728 | 1.07 | 17 (13, 22) | 666 | 14570 | 1.19 | 17 (11, 21) | 1.11 (0.93, 1.32) | 0.254 |
| URTI | 154 | 3720 | 1.08 | 17 (13, 22) | 663 | 14553 | 1.18 | 17 (11, 21) | 1.10 (0.92, 1.32) | 0.275 |
| LRTI ^b^ | 22 | 4693 | 0.12 | 15 (13, 23) | 93 | 18504 | 0.13 | 15 (11, 21) | 1.07 (0.66, 1.72) | 0.790 |
| HLRTI | 10 | 4736 | 0.05 | 22 (15, 25) | 45 | 18657 | 0.06 | 16 (13, 22) | 1.14 (0.57, 2.31) | 0.706 |

^a^ p-values from a cox proportional hazards model. Jackknife estimation of standard errors was used to account for repeated events within the same infant

^b^ Non-hospitalized and hospitalized cases of lower respiratory tract infection

**Figure**

## **Figure S1. Kaplan-Meier curve for not experiencing a first microbiologically confirmed ARI in the first 26 weeks of age among infants enrolled from birth (n = 829), by supplementation group**


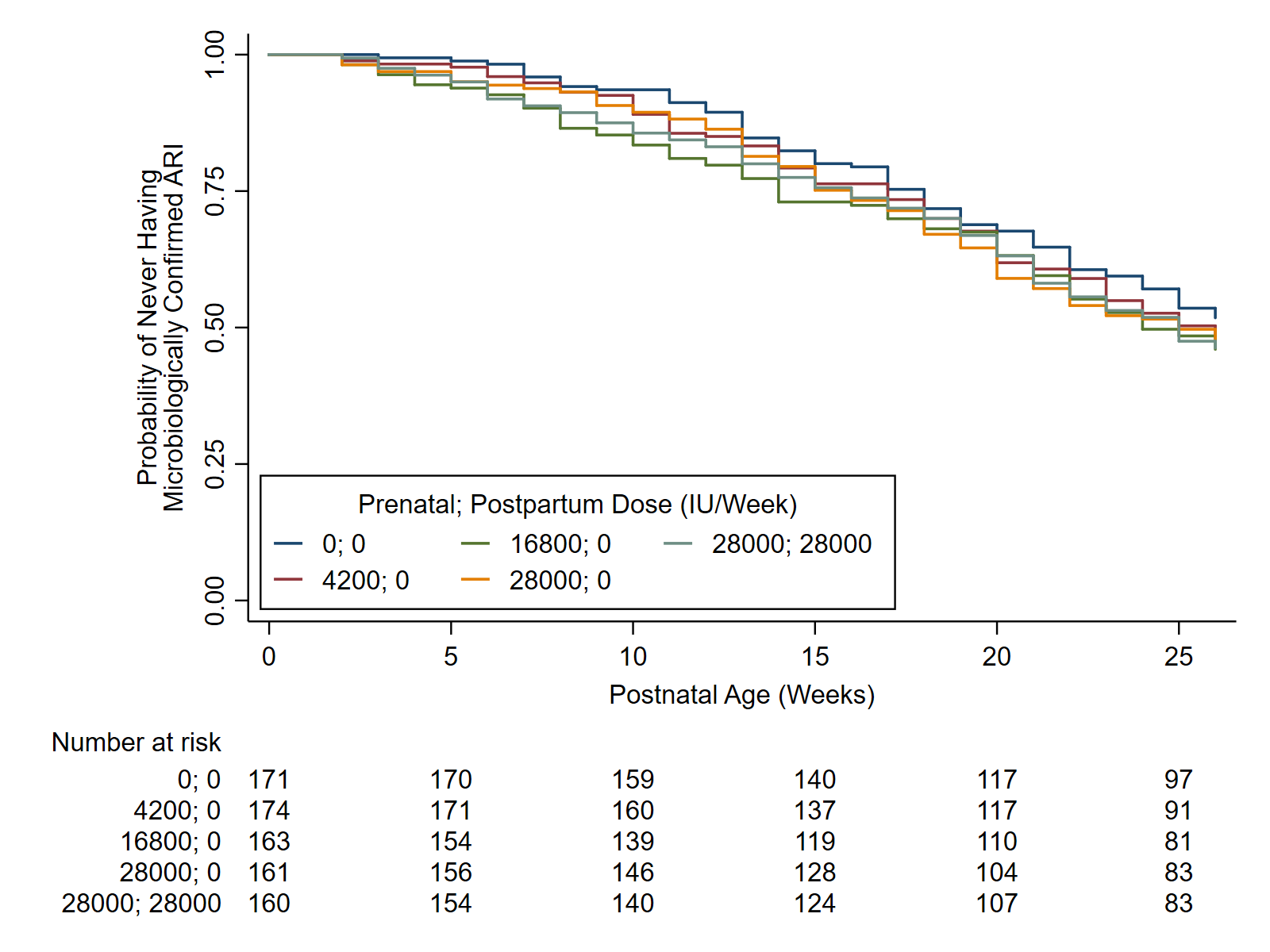

Supplement: piab032_suppl_Supplementary_Material [file piab032_suppl_supplementary_material.docx]
